# Lateral septum-lateral hypothalamus circuit dysfunction in comorbid pain and anxiety

**DOI:** 10.1038/s41380-022-01922-y

**Published:** 2023-01-16

**Authors:** Di Wang, Xiangyu Pan, Yu Zhou, Zifeng Wu, Kunpeng Ren, Hanyu Liu, Chaoli Huang, Yumei Yu, Teng He, Xiao Zhang, Ling Yang, Hongxing Zhang, Ming-Hu Han, Cunming Liu, Jun-Li Cao, Chun Yang

**Affiliations:** 1 Department of Anesthesiology and Perioperative Medicine, The First Affiliated Hospital of Nanjing Medical University, Nanjing, 210029 China; 2 Jiangsu Province Key Laboratory of Anesthesiology, Jiangsu Province Key Laboratory of Anesthesia and Analgesia Application Technology, Xuzhou Medical University, Xuzhou, 221000 China; 3 Department of Anesthesiology, The Affiliated Wuxi NO. 2 People’s Hospital of Nanjing Medical University, Wuxi, 214000 China; 4 Department of Cardiology, The Third Affiliated Hospital of Soochow University, Changzhou, 213000 China; 5 Department of Mental Health and Public Health, Faculty of Life and Health Sciences, Shenzhen Institute of Advanced Technology, Chinese Academy of Sciences, Shenzhen, 518000 China

**Keywords:** Neuroscience, Physiology

## Abstract

Pain and anxiety comorbidities are a common health problem, but the neural mechanisms underlying comorbidity remain unclear. We propose that comorbidity implies that similar brain regions and neural circuits, with the lateral septum (LS) as a major candidate, process pain and anxiety. From results of behavioral and neurophysiological experiments combined with selective LS manipulation in mice, we find that LS GABAergic neurons were critical for both pain and anxiety. Selective activation of LS GABAergic neurons induced hyperalgesia and anxiety-like behaviors. In contrast, selective inhibition of LS GABAergic neurons reduced nocifensive withdrawal responses and anxiety-like behaviors. This was found in two mouse models, one for chronic inflammatory pain (induced by complete Freund’s adjuvant) and one for anxiety (induced by chronic restraint stress). Additionally, using TetTag chemogenetics to functionally mark LS neurons, we found that activation of LS neurons by acute pain stimulation could induce anxiety-like behaviors and vice versa. Furthermore, we show that LS GABAergic projection to the lateral hypothalamus (LH) plays an important role in the regulation of pain and anxiety comorbidities. Our study revealed that LS GABAergic neurons, and especially the LS^GABAergic^-LH circuit, are a critical to the modulation of pain and anxiety comorbidities.

## Introduction

Chronic pain is a thorny medical problem, with the current treatment measures failing to achieve satisfactory results [1]. Chronic pain is often accompanied by several mental diseases or abnormalities, of which anxiety is prominent [2, 3]. Importantly, anxiety aggravates pain symptoms, possibly causing a vicious cycle between pain and anxiety [4]. Unfortunately, most previous investigations of pain and anxiety were carried out independently. Only a few studies addressed the interactions between pain and anxiety at cellular, network and synaptic levels [5]. Therefore, finding effective therapeutic strategies for the comorbidity of pain and anxiety poses a major research challenge and promises significant medical and social impacts.

The lateral septum (LS) is implicated as a hub that regulates affective behaviors, such as reward, feeding, anxiety, fear, sociability, and memory [6–8]. It is well known that LS neurons are predominantly GABAergic, with only a small population of glutamatergic cells in the most ventral region [9]. The LS has been traditionally divided into three main divisions based on their distinct major afferent and efferent connections [7]. Descending LS GABAergic projections to several brain regions, including the nucleus accumbens (NAc), vertical limb of the diagonal band (VDB), horizontal limb of the diagonal band (HDB), dorsomedial hypothalamus (DM), lateral preoptic area (LPO), ventromedial hypothalamus (VMH), lateral hypothalamus (LH), hippocampus (HPC), ventral tegmental area (VTA), and periaqueductal gray (PAG), are involved in the central regulation of sensory or emotional aspects of behavior [10–13]. Intriguingly, LS neurons receive nociceptive inputs from the thalamus and somatosensory cortices, and fear and/or anxiety-related projections from the hippocampus [14, 15]. Given this unique feature that allows LS neurons to integrate sensory inputs and emotion signaling, we hypothesized that LS was a brain region with potentially important roles in pain-anxiety interactions.

The lateral hypothalamic area (LH) is a vital controller of arousal, feeding, and metabolism, integrating sensory information about the world and the body [8, 16]. The LH had been identified as a brain region responsive to pain stimuli and chronic pain-induced maladaptive anxiety that is capable of controlling pain-related behavioral responses by feeding [17, 18]. Interestingly, the inhibitory LS to LH circuit can suppress feeding [16]. However, the causal relationship between the adaptation of LS circuits and the pathology of comorbid pain and anxiety is unknown. Given the fact that both LS and LH were strongly linked to both anxiety and pain, we further hypothesized that the LS-LH circuit is probably involved in the pathologic causes of pain and anxiety comorbidity.

We address the above hypotheses in behavioral and neurophysiological experiments in mice using a multipronged approach combining methods of viral tracing, fiber photometry, electrophysiology, optogenetics, and chemogenetic and TetTag chemogenetic manipulations. We traced the brain-wide connection of LS, demonstrated the role of LS GABAergic neurons in regulating pain and anxiety behavior, and dissected the functional organization of the LS^GABAergic^ -LH circuit in pain and anxiety comorbidities. We explored the principles of LS involvement in pain and anxiety adaptation in two male mouse models. The first one was a model of chronic inflammatory pain, induced by the administration of complete Freund’s adjuvant (CFA). The second was a model of anxiety, induced by chronic restraint stress (CRS). Based our findings, we propose that the GABAergic neurons of LS, and especially the LS^GABAergic^ -LH circuit, are critical for the comorbidity of pain and anxiety and its treatment.

## Materials and methods

### Animals

Male C57BL/6 mice (Beijing Charles River Laboratory Animal Breeding Co. Ltd., China) and female and male *Vgat-ires-Cre* transgenic mice (*Vgat-ires-Cre:* Slc32a1tm2(Cre)Lowl/J, RRID: 016962; The Jackson Laboratory, ME, USA) were purchased at age 8–12 weeks and housed 4–5 per cage. The animals were maintained at a controlled temperature (24 °C ± 2 °C) with 12:12 h light/dark cycle (lights on at 07:00) and ad libitum access to food and water. Only adult male mice were used in the behavioral experiments. Adult male and female mice with a similar distribution were used for adeno-associated virus (AAV) and herpes simplex virus (H129) anterograde tracing and in vivo electrophysiological experiments.

All procedures were approved by the Institutional Animal Care and Use Committee of Nanjing Medical University and Xuzhou Medical University and were performed in accordance with the National Institutes of Health Guide for the Care and Use of Laboratory Animals.

### Statistical analysis

Prior to further analysis, each dataset was subjected to a normality test using the Shapiro–Wilk test. For datasets that were normally distributed, parametric tests of the variance and group differences (paired, unpaired t-tests, one-way or two-way ANOVA with LSD post hoc test) were used. Otherwise, the non-parametric Wilcoxon matched-pairs signed rank test, Mann Whitney Rank Sum Test, and the Kruskal Wallis One Way ANOVA on ranks with Uncorrected Dunn’s test were used. For the analysis of correlations between the mechanical withdrawal threshold and OFT/EPM anxiety behaviors, we used the Pearson correlation for normally distributed data and the Spearman rank correlation otherwise. Statistical tests were performed using statistical software packages (MATLAB2017a, MathWorks.Inc, USA or GraphPad Prism 9, GraphPad Software.Inc, USA). Data are presented as the mean ± SEM. Statistical significance was set at three levels (*p < 0.05, **p < 0.01, ***p < 0.001)

Detailed information about the materials and methods is described in the supplementary material.

## Results

### Chronic inflammatory pain and anxiety interact

**Fig. 1:**
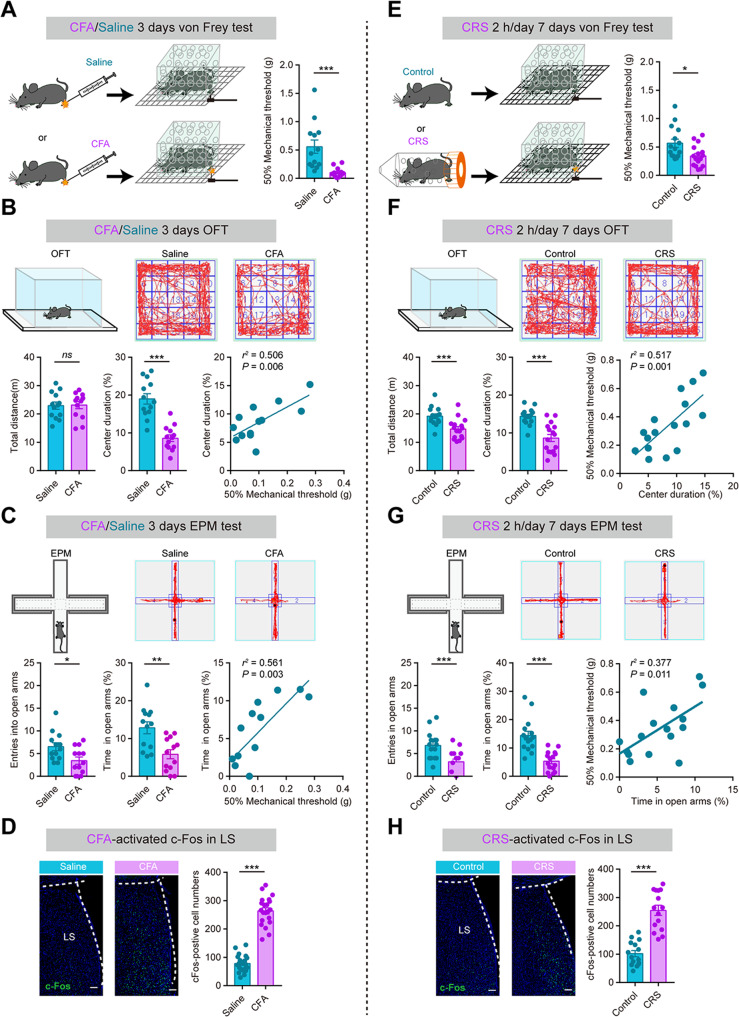
Chronic pain induces anxiety-like behaviors and chronic restraint stress (CRS) aggravates pain behaviors. **A**–**D** Data representative for the chronic pain model (CFA). **A** Withdrawal threshold of hind paw in response to von Frey hair stimulation under chronic inflammatory pain. **B** Open field test (OFT). (Top) Representative locomotion traces. (Bottom, left) Distance traversed. (Bottom, middle) percentage of time spent in the central zone. (Bottom, right) Correlation between withdrawal threshold and time spent in the central zone. **C** Elevated plus maze **(**EPM) test. (Top) Representative exploration traces. (Bottom, left) Entries made into arms. (Bottom middle) percentage of time in spent in open arms. (Bottom, right) Correlation between withdrawal threshold and time spent in EPM open arms. **D** (Left) c-Fos expression levels in LS are elevated in CFA over those in the control. Scale bar, 100 μm. **E**–**H** Data representative for the anxiety model (CRS) shown similarly to (**A**–**D**). *P < 0.05, **P < 0.01, ***P < 0.001. *ns*, no significant difference (*P* > 0.05). Data are presented as the means ± SEM. For further details of statistical data analysis see Table S1.

Chronic inflammatory pain was induced by CFA as previously reported [3], and von Frey monofilaments were used to assess mechanical hyperalgesia. Three days after CFA injection, the mice exhibited a decrease in the paw withdrawal threshold (Fig. 1A). They also spent less time in the center in the open field test (OFT) (Fig. 1B) and made fewer entries and spent less time in the open arms in the elevated plus maze (EPM) test (Fig. 1C). These findings indicate that chronic pain elicited anxiety-like behaviors. The paw withdrawal threshold was positively correlated with the time spent in the OFT center and EPM open arms (Fig. 1B, C). Locomotor activity, measured by the total distance traversed in OFT, was not significant different between the CFA and saline groups (Fig. 1B). We used the tail-suspension test (TST) and forced-swim test (FST) to measure behavioral despair/helplessness. The mice in the CFA group, when compared with those in the saline control, showed no change in the immobility time (Fig. S1A). These findings demonstrate that CFA mice, a chronic inflammatory pain mouse model, successfully develop an anxiety-like phenotype without enhanced behavioral despair.

We used the same experiments to investigate the effect of CRS on behaviors. To induce anxiety-like behavior in the CRS mice, animals were placed in well-ventilated 50-ml falcon tubes for 2 h (between 10:00 a.m. and 12:00 a.m.) daily for 7 consecutive days (Fig. 1E). On day 8, CRS mice started to spend shorter time in the OFT center field and covered shorter distances than mice in the control group (Fig. 1F). EPM test results were consistent with the OFT results: CRS mice spent significantly less time in the open arms and entered the open arms less frequently (Fig. 1G). The pain threshold was lower in the CRS than the control mice (Fig. 1E). Although CRS mice did not have as low a pain threshold as CFA mice (CFA *vs*. CRS: 0.10 ± 0.02 *vs*. 0.34 ± 0.05 g). Correlation analysis shown the time spent in the OFT center or EPM open arms was positively correlated with the paw withdrawal threshold (Fig. 1F, G). The immobility time of mice in TST/FST was not affected by CRS conditioning (by day 7 day) (Fig. S1B). These findings suggest that CRS has a potential to aggravate pain behaviors. Interestingly, both CFA and CRS mice lost body weight (Fig. S2), suggesting that both pain and anxiety may induce abnormal feeding behaviors with effects on the metabolic process that are harmful to the animal.

### LS GABAergic neurons are activated by comorbid pain and anxiety

The LS has been implicated in emotional regulation, particularly in anxiety processing [19]. With this in mind, we studied LS neuron activity in CFA and CRS mice by measuring the levels of c-Fos expression in LS. Compared with saline mice, CFA mice expressed c-Fos in the LS at elevated levels after CFA injection (Fig. 1D). A similar effect was found in CRS mice, with the number of c-Fos positive cells in their LS region significantly greater than in control mice (Fig. 1H). Consistent with the c-Fos data, in vivo electrophysiological recordings revealed increased LS neuronal firing immediately after the delivery of light pain stimulus (Fig. S3A–C).

**Fig. 2:**
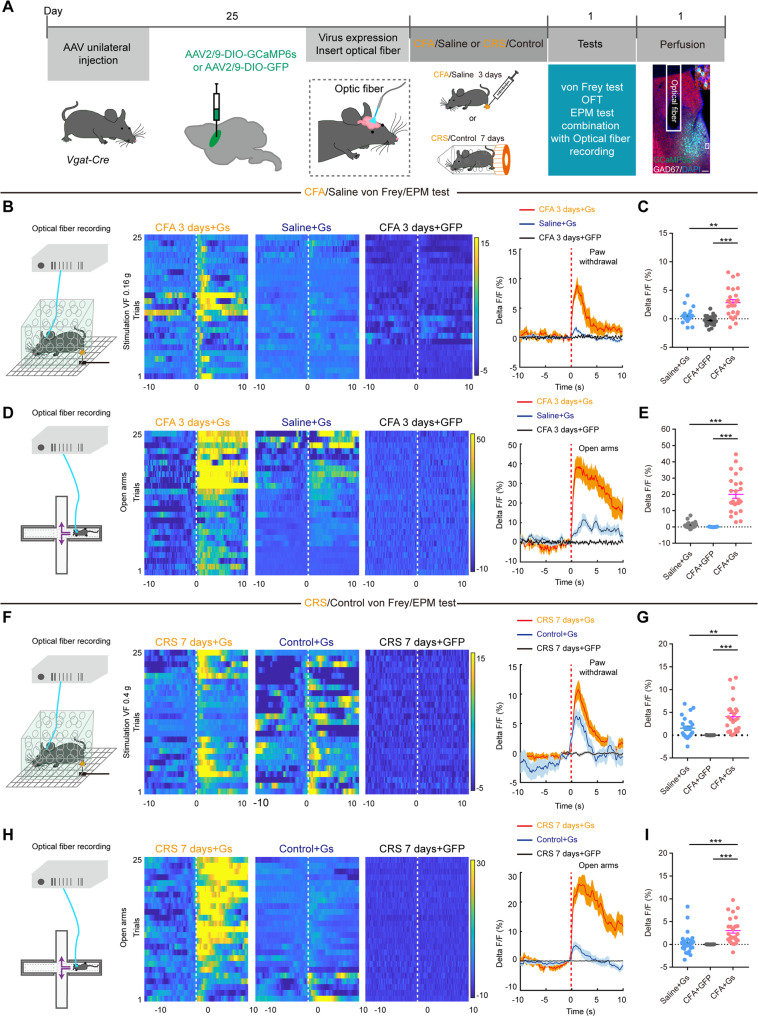
GABAergic neurons in LS are activated in comorbid chronic pain and anxiety. **A** Schematics of the data acquisition system used for Ca^2+^ imaging with fiber photometry in combination with recording behaviors tests (von Frey, OFT, and EPM). Also shown is GCaMP6s expression in LS. Scale bar, 100 μm. **B**, **D, F**, **H** (Left) Schematics of fiber photometry recording in freely moving mice simultaneously receiving punctate mechanical stimulation (von Frey filament, CFA: 0.16 g and CRS: 0.4 g) or entering EPM open arms. Heatmap and average Ca^2+^ transients of LS GABAergic neurons in mice receiving von Frey punctate stimulation or entering open arms. (**C, E, G**, **I**) Statistics of the average Ca^2+^ fluorescence signaling changes across mice receiving 0.16/0.4 g von Frey stimulation or time-locked to the entry into the EPM open arms. **P < 0.01, ***P < 0.001. Data are presented as the means ± SEM. For further details of statistical data analysis see Table S1.

Next, we proceeded to quantify the physiological activity of selectively targeted LS GABAergic neurons during nocifensive and emotional behaviors. To do this, we infused Cre-dependent adeno-associated virus (AAV) vector expressing a Ca^2+^-sensor protein (GCaMP6s) in LS GABAergic neurons via stereotaxically targeted microinjection and measured changes in GCaMP6s fluorescence (Fig. 2A). After 25 days rest, CFA and CRS models were conducted. Increased LS GABAergic neuronal activity was recorded by in vivo fiber photometry recording following pain stimulation by von Frey filaments applied to the contralateral hind paw of CFA (Fig. 2B, C) and CRS mice (Fig. 2F, G). After three days of CFA injections, OFT or EPM tests were conducted with simultaneous recordings of LS GABAergic neuronal activity using position-synchronized in vivo calcium imaging. In the OFT, CFA and CRS mice preferred walking along the wall, and the Ca^2+^ transient activity in the LS GABAergic neurons were increased as CFA and CRS mice moved from the surrounding of the wall to the central location (Fig. S3D–G). CFA and CRS mice spent less time exploring in the open arms and more time in the closed arms of EPM (Fig. 1C, G). Meanwhile, the average Ca^2+^ activities in LS and the variation rate of Ca^2+^ fluorescence in the LS GABAergic neurons significantly increased during times when the CFA and CRS mice engaged in open-arm exploration, but not closed-arm exploration (Fig. 2D, E, H, I). Together, these results represent strong evidence that the comorbidity of pain and anxiety activate GABAergic neurons in LS.

### Activation of LS GABAergic neurons triggers the comorbidity of pain and anxiety

**Fig. 3:**
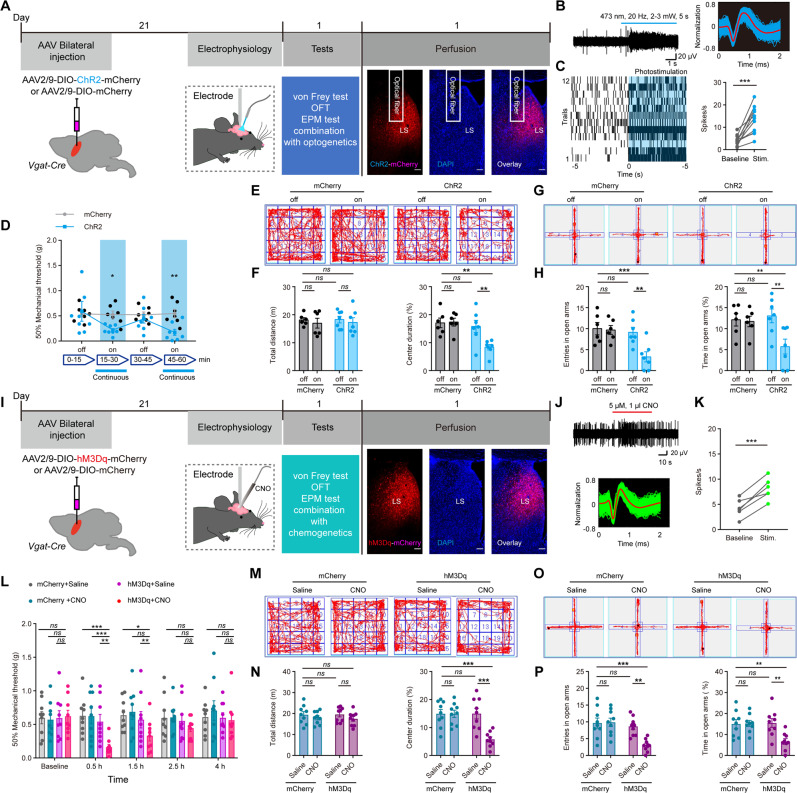
Selective activation of LS neurons decreased pain threshold and induced anxiety-like behaviors. **A** Schematics of the experiment. Coronal section showing ChR2-mCherry expression and optical fiber insertion in the LS. Scale bar, 100 μm. **B** (Left) Sample traces of in vivo photo-tagged single unit spike recordings and (Right) normalized spikes waveforms. **C** Example rasters of spike trains recorded while a 20 Hz train of light pulses (started at time 0) ChR2-optogenetically activated GABAergic LS neurons. (Right) Firing activity of neurons upon ChR2-optogenetic stimulation. The behavioral effects of optogenetic activation of LS GABAergic neurons were assessed by (**D**) mechanical pain threshold, **E** OFT exploration, **F** (Left) total distance traversed in the OFT, (Right) time spent in central of OFT, **G** EPM exploration, **H** (Left) Total entries in arms, and (Right) time spent in open arms. **I** Experimental scheme of chemogenetic virus injection and behavioral tests. Coronal section showing the expression of hM3Dq-mCherry in the LS of *Vgat-Cre* mice. Scale bar, 100 μm. **J**–**K** Firing activity of neurons upon CNO-stimulated hM3Dq-expressing LS neurons. Data presented as in (**B**–**C**). **L**–**P** The behavioral effects of chemogenetic manipulation of LS GABAergic neurons were assessed exactly as in (**D**–**H**), with similar result. *P < 0.05, **P < 0.01, ***P < 0.001. *ns*, no significant difference (*P* > 0.05). Data are presented as the means ± SEM. For further details of statistical data analysis see Table S1.

Since the association between LS GABAergic neuron hyperactivity and the comorbidity of pain and anxiety behavior had been well established, we tested whether the activation of LS GABAergic neurons was sufficient to induce comorbid behavior. In animals that expressed ChR2-mCherry spatially restricted to the LS, the optical fiber was implanted in the center of virus expression (Fig. 3A). We used electrophysiology recordings to ascertain the efficacy of neuronal activity under optogenetic activation (20 Hz, 5 ms pulse, laser 473 nm) of GABAergic neurons in LS (Fig. 3B, C). We found that the optogenetic activation of LS GABAergic neurons accompanied by a time-dependent reduction of pain threshold (Fig. 3D), and a significant reduction in exploration time spent in the central area of OFT, but no change in locomotor activities in OFT (Fig. 3E, F). Optogenetic LS activation similarly decreased the number of open-arm entries and the time spent in the open arms in the EPM test (Fig. 3G, H).

In an independent chemogenetic manipulation experiment (Fig. 3I), we bilaterally infected LS neurons of *Vgat-Cre* mice with a hM3Dq-mCherry (Designer Receptors Exclusively Activated by Designer Drugs, DREADDs) virus vector. This allows the rapid selective excitation of hM3Dq-mCherry expressing neurons via the activation of hM3Dq receptors with clozapine-N-oxide (CNO). To do this, we intraperitoneally injected the animals with CNO (2 mg/kg). Again, we used electrophysiology recordings to ascertain successful activation (as measured by increased firing) of LS GABAergic neurons after CNO treatment (Fig. 3J, K). Then we found the paw withdrawal threshold was decreased at 0.5 h and 1.5 h after CNO injection (Fig. 3L) and the anxiety-like behaviors were also induced with CNO administration (Fig. 3M–P). We found that mice with CNO administration significantly reduced the exploration time in the central area of OFT, but showed no change in locomotor activities (Fig. 3M, N). CNO-mediated LS GABAergic neurons activation decreased the time spent in the open arms and the number of open-arm entries (Fig. 3O, P). In summary, both direct optogenetic and chemogenetic stimulation of LS GABAergic neurons enabled the phenotype onset of the comorbidity of pain and anxiety.

To further explore the function of pain/anxiety-related neurons in LS on anxiety/pain, we set up a TetTagging system using AAV vectors so that we could target pain- or anxiety-related LS neurons. Because of the size constraints of the AAV genome, we needed to generate two AAV viruses, one that contained the *P*cfos-tTA transgene, and another that contained the tet-operator promoter (PTRE-tight) linked to an hM3Dq-mCherry receptor reading frame (Fig.S4A). We used this approach to set up the TetTag system and kept it suppressed for 28 days with doxycycline supplied in the drinking water of the animals before behavioral experiments were undertaken. Doxycycline blocks tTA from activating its target promoter, PTRE-tight. However, when doxycycline was removed for the 2 days of the behavioral experiments, increased LS neural activity, such as that occurring under acute pain stimulation with the 1.0 g von Frey filament or during acute restraint stress, can drive the c-Fos promoter–linked tTA expression, which in turn can activate hM3Dq-mCherry expression in LS, making it possible to selectively label functionally activated LS neurons (Fig. S4B). Before the intraperitoneal injection of CNO or saline, there was no difference in the pain threshold or anxiety behavior between the saline and CNO groups (Fig. S4C–E). However, upon CNO injection, and the associated chemogenetic activation of the pain-related neurons in LS caused measurable changes in the animals’ behavior, with a decrease in the total distance covered in the OFT test and a reduction in the exploration time spent in the central area of OFT (Fig. S4C). Meanwhile, the same manipulation led to shorter exploration of the open-arm without altering the frequency of visits to the open arm (Fig. S4D). Similar activation of anxiety-related neurons led to a decrease in the mechanical pain threshold in mice (Fig. S4E). Based on these results, we speculated that pain and anxiety processing networks shared a subpopulation of LS neurons. This scenario would explain the observed LS regulation of the comorbidity of pain and anxiety.

### Ablation or inhibition of LS GABAergic neurons promotes resilience to the comorbidity of pain and anxiety

To further characterize the precise role of LS GABAergic neurons in the comorbidity of pain and anxiety behaviors, we performed cell ablation and optogenetic/chemogenetic manipulations of these neurons. Consistent with its apoptosis-inducing effect, taCasp3 gradually eliminated GABAergic neurons in the LS, resulting in an average loss of neuronal fluorescence intensity 3 weeks after virus injection (Fig. S5A–C). Compared with the control mice, taCasp3-injected CFA mice developed a remarkable increase in pain threshold on days 7, 14, and 21 (Fig. S5D), spent more time in the central area of OFT on days 7, 14 (Fig. S5E), and increased the time spent in and the number of entries into the EPM open arms on days 7, 14, and 21 (Fig. S5F), with no change in their locomotor activities (Fig. S5E). Furthermore, we found a positive correlation between the size of LS lesion and pain thresholds (Fig. S5G) or time spent in the open arms (Fig. S5H). Additionally, the behavioral tests of anxiety demonstrated that the ablation of LS GABAergic neurons significantly blocked the anxiogenic effects of CRS mice including increased total distance, time in center of OFT test and entries and time in open arms of EPM test (Fig. S5I–K).

**Fig. 4:**
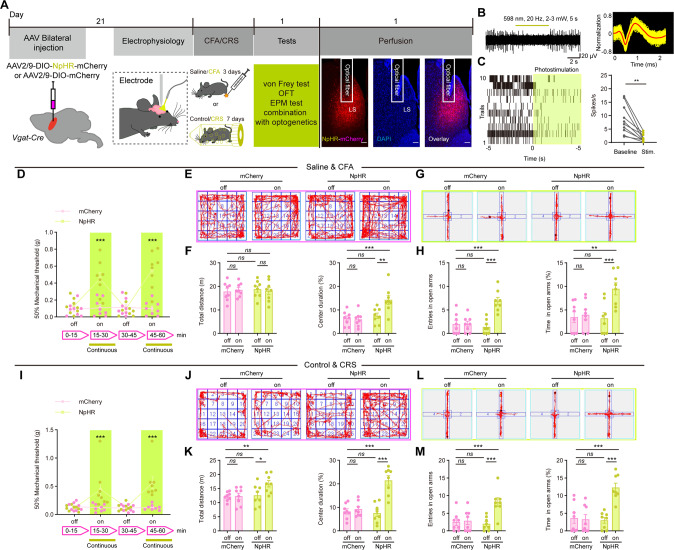
Inhibition of LS GABAergic neurons reverse the comorbidity of chronic pain and anxiety. **A** Schematics of the experiment. Coronal section showing NpHR-mCherry expression and optical fiber insertion in the LS. Scale bar, 100 μm. **B** (Left) Sample trace of in vivo photo-tagged extracellularly recorded spike trains and (Right) normalized single unit spikes waves. **C** (Left) Rastergrams of spiking activity recorded for LS GABAergic neurons during their targeted optogenetic inactivation. (Right) Firing activity of neurons upon NpHR-optogenetic stimulation. The behavioral effects of optogenetic inhibition of LS neurons in CFA mice: **D** Mechanical pain sensitivity during light-off and light-on sessions. **E** OFT track traces of CFA mice infected with mCherry or NpHR-mCherry. **F** OFT (Left) total distance and (Right) percentage of time in center. **G** EPM track traces. **H** (Left) number of entries into and (Right) percentage of time spent in EPM open arms. **I**–**M** Data for the same experiments as shown in (**D**–**H**) but for the CRS model. *P < 0.05, **P < 0.01, ***P < 0.001. *ns*, no significant difference (*P* > 0.05). Data are presented as the means ± SEM. For further details of statistical data analysis see Table S1.

The findings discussed above suggest that LS GABAergic hyperactivity is a trait of the comorbidity of pain and anxiety, and we hypothesized that inhibiting LS GABAergic neurons activity (by means of experimental manipulations) would have the opposing effect, with a measurable increase in the animals’ pain threshold and resilience to the anxiety-like behavior, in CFA and CRS mice. For such manipulations, we proceeded to use optogenetic (Fig. 4) or chemogenetic (Fig. S6) inactivation. We bilaterally infected LS neurons with AAV2/9-DIO-NpHR-mCherry (for infected neurons to express halorhodopsin, a yellow-light-sensitive chloride channel that silences the firing activity of the cell by hyperpolarization)/AAV2/9-DIO-mCherry or AAV2/9-DIO-hM4Di-mCherry (DREADDs, inhibition of neuronal activity)/ AAV2/9-DIO-mCherry of *Vgat-Cre* mice. We verified that the infections were restricted to the LS without leakage into other brain regions, and both could inhibit neuronal excitability (Fig. 4A–C; Fig. S6A, B). Selective optogenetic inhibition of GABAergic neurons in LS increased the CFA and CRS animals’ pain threshold (Fig. 4D, I), and diminished anxiety-like behavior in CFA and CRS model mice, such as increasing center duration and not affecting the total distance of OFT test, and both increasing entries and time in open arms of EPM test in CFA mice (Fig. 4E–H), increasing total distance, center duration, entries and time in open arms of OFT and EPM tests in CRS mice (Fig. 4J–M). Chemogenic manipulation had a similar effect in both of CFA and CRS mice. After the animals were injected with CNO (2 mg/kg, i.p.), the pain threshold was significantly increased at post-injection times 0.5 h, 1.5 h, and 2.5 h (CFA) or 0.5 h and 1.5 h (CRS), (Fig. S6C, H) and this was accompanied by anxiolytic behaviors in both groups including increasing total distance, center duration, number of entries and time in open arms of OFT and EPM tests in CFA/CRS mice (Fig. S6D–G, I–L).

### LS GABAergic neurons project broadly to pain and anxiety processing centers in the brain

**Fig. 5:**
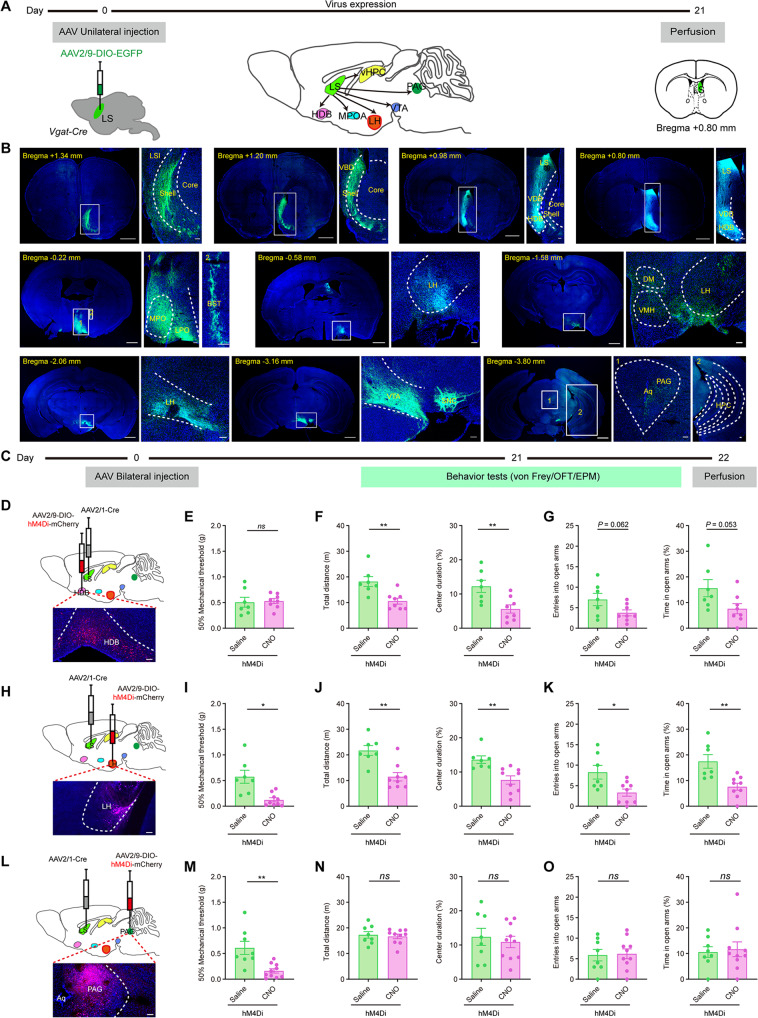
Mapping and chemogenetic activation of axonal projections from LS GABAergic neurons. **A**, **B** The distribution pattern of the LS GABAergic projection. **A** Schematics and timeline of antegrade virus (AAV2/9-DIO-EGFP) tracing. **B** Schematic summary of mapped recipient brain regions of LS projection fibers with the distribution pattern from whole-brain tracing. **C**–**O** By using chemogenetic manipulation, LS GABAergic projections to HDB, LH, PAG were indirectly activated in behavioral experiments. **C** Experimental scheme of virus injections and behavior tests. Schematics of the stereotaxic surgery targeting the (**D)** LS-HDB, (**H)** LS-LH, and (**L**) LS-PAG circuits. Coronal sections illustrating hM4Di mCherry-positive HDB, LH and PAG neurons at the injection site. Scale bar, 100 μm. **E**–**G** Behavioral results for LS-HDB activation. **E** Withdrawal threshold of the hind paw measured with the von Frey test. **F** OFT (Left) total distance and (Right) percentage of time in center. **G** EPM (Left) total entries in arms and (Right) time in open arms. **I**–**K** Same as (**E**–**G**) but for LS-LH activation. **M**–**O** Same as (**E**–**G**) but for LS-PAG activation. *P < 0.05, **P < 0.01. *ns*, no significant difference (*P* > 0.05). Data are presented as the means ± SEM. For further details of statistical data analysis see Table S1. DM, dorsomedial hypothalamus; HDB, horizontal limb of the diagonal band; HPC, hippocampus; LH, lateral hypothalamus; LPO and MPO, lateral and medial preoptic area; NAc, nucleus accumbens; PAG, periaqueductal gray; VDB, vertical limb of the diagonal band; VMH, ventromedial hypothalamus; VTA, ventral tegmental area.

To further understand how LS GABAergic neurons exert regulatory effects on the comorbidity of pain and anxiety, we traced the axonal projections of LS GABAergic neurons. First, the anterograde vector tracers AAV2/9-DIO-EGFP or H129-ΔTK-tdT were injected into LS of *Vgat-Cre* mice, and the expression of virus in the whole brain was observed (Fig. 5A and Fig. S7A). The downstream targets of LS GABAergic neurons included the NAc, VDB, HDB, DM, LPO, VMH, LH, HPC, VTA, and PAG (Fig. 5B; Fig. S7B, C). Most of these regions had been previously implicated in sensory or emotional processing. For example, both the HDB and LH are thought play important roles in the regulation of emotions. The PAG is a known as important hub that processes pain signals in descending pathways.

### Activation of LS projection induces comorbid pain and anxiety behavior in naïve mice

Different subregions of LS, such as the dorsal and ventral parts, are known to play different roles in the regulation of emotion and other related behaviors [20]. We speculated that these differences might also be reflected in the differences in the anatomical projection targets (i.e., downstream brain regions) associated with the distinct LS subpopulations. To examine the differences in the downstream projection of different LS subregions, we injected anterograde vectors expressing EGFP and mCherry into the dorsal and ventral LS, respectively (Fig. S8A, B). These experiments revealed obvious differences in the brain-wide distribution of the downstream projections between the two subregions of LS. The projection of dorsal LS tends to HDB, LH, VTA et al. while the projection of ventral LS tends to DM, VMH and other brain regions (Fig. S8C, D).

Next, we investigated the role of the different LS projection pathways in pain and anxiety regulation. In selected known target regions of LS projections, we bilaterally injected a retrograde virus vector of a Cre recombinant enzyme (Retro-AAV-Cre) into HDB, LH, PAG, medial preoptic area (MPOA), ventral HPC (vHPC) and VTA, and we also injected a Cre-dependent chemogenetic vector (AAV2/9-DIO-hM3Dq-mCherry) into the bilateral LS (Fig. S9A, E, I; Fig. S10A, E, I) for the selective chemogenetic activation of LS projection neurons. Three weeks after full expression of the virus in the LS, intraperitoneal injection of CNO (2 mg/kg) activates LS projection. We then assessed OFT and EPM testing behaviors. We observed activation of LS projections to HDB and LH reduced time spent in OFT center, EPM open arms and entries in open arms; The projection of LS to HDB decreased the total distance in OFT, but the projection of LS to LH did not affect the total distance (Fig. S9C, D, G, H). Interestingly, the activation of the LS to LH projection had an effect on the pain threshold, the projection of LS to HDB did not change the pain threshold (Fig. S9B, F). The activation of LS projections to PAG also reduced pain thresholds but did not induce anxiety-like behavior (Fig. S9J–L). Chemogenetic activation of the MPOA-projecting, vHPC-projecting, and VTA-projecting LS neurons had no effect on the pain threshold or anxiety behaviors (Fig. S10A–L).

However, we could not fully distinguish the specific roles of the HDB-, LH- and PAG-projecting LS neurons in the regulation of pain and anxiety using the neural circuit construction method described above. To overcome this limitation and to obtain more convincing evidence, C57BL/6 mice were infused with AAV2/1-Cre into the LS, which could anterograde transport from the neuronal cell body to the axon terminal and then across the monosynapse into its projecting neurons, and AAV2/9-DIO-hM4Di-mCherry into the HDB, LH and PAG (Fig. 5C, D, H, L). Through the above circuit construction and with the intraperitoneal injection of CNO (2 mg/kg), the HDB-, LH- and PAG-projecting LS neurons were indirectly activated. The results showed that activation of the neural projection from LS to HDB and to LH triggered anxiety-like behavior including decreased exploring time in the center and total distance in the OFT (Fig. 5F, J) and reduced the number of times and time into the open arm (Fig. 5G, K). The results of von Frey stimulation also showed that activation of neural projection from the LS to LH and PAG neurons induced hyperalgesia (Fig. 5I, M). Activation of LS projection to HDB did not affect the mechanical pain threshold in mice (Fig. 5E). We found that activation of neural projection from the LS to PAG neurons displayed no anxiety-like behaviors in the EPM and OFT tests (Fig. 5N, O). These findings are further confirmation of the remarkable functional differences of the HDB-, LH-, and PAG-projecting subpopulations of LS GABAergic neurons in the regulation of anxiety and pain.

To determine whether these divergent projections originate from different subsets of LS neurons or reflect collateral projections from the same LS population, we performed simultaneous retrograde tracing from the 3 target regions using fluorescent labels of different color. After injection of Retro-AAV-hSyn- expressing mCherry (red), EGFP (green) and mTagBFP-3XFlag (blue) into the HDB, LH and PAG, respectively (Fig. S11A, B), we found strong expression of mCherry, EGFP and mTagBFP-3XFlag in the LS. The expression of mCherry and mtagBFP-3xFlag partially overlapped with EGFP (46.19% and 20.65%), but little overlap between mCherry and mTagBFP-3XFlag (12.78%) (Fig. S11C, D). These experiments suggest that the HDB-, LH - and PAG -projecting LS neurons form largely distinct subpopulations.

### Activity of HDB-, LH- and PAG-projecting LS neurons mediates anxiety-like behavior

Above we demonstrated how LS neuron activity affected the behavior of the animals. We next determined whether the activity of the HDB-, LH - and PAG -projecting LS neurons would change in response to noxious stimuli or anxiety behaviors. First, C57BL/6 mice were injected with AAV2/9-DIO-GCaMP6s into the HDB, LH or PAG, and AAV2/1-Cre into the LS. An optic-fiber cannula was implanted in the brain of the animal over the HDB, LH or PAG. We used mice of the CFA model to induce comorbid pain and anxiety behaviors. A 3-day CFA conditioning was conducted, followed by the von Frey, OFT and EPM tests combined with simultaneous monitoring of neuronal activity in the HDB, LH or PAG using position-synchronized in vivo fiber photometry recordings (Fig. S12A). The green fluorescence signals observed in the LS projection areas verified that the AAV2/1-Cre vector crossed the synapse at the LS axon terminals in HDB, LH, and PAG and helped the expression of GCaMP6s in the soma of HDB, LH or PAG neurons (Fig. S12B, F, J).

Mechanical stimulation inhibited calcium signaling in HDB, LH and PAG neurons (Fig. S12C, G, K). The position-synchronized in vivo calcium recording revealed that CFA mice exhibited significant inactivation of GCaMP6s activity following emotional stress exposure of HDB, LH neurons monosynaptically innervated by GABAergic LS projections (Fig. S12D, E, H, I). Calcium signal in PAG was responsive to EMP test, but not to OFT test (Fig. S12L, M). The above results indirectly reflect that mechanical pain stimulation, OFT and EPM test can activate the GABAergic neurons in LS.

Notably, above studies have shown that the LS^GABAergic^-LH circuit played an importance role in the regulation of comorbid pain and anxiety. To further establish a functional link between the LS and LH, ChR2-mCherry was virally expressed in LH neurons that were the monosynaptic targets of axon projections originated from LS GABAergic neurons (Fig. S13A). In vivo electrophysiological recordings showed that blue laser pulses inhibited LH neurons firing in a time-locked manner (Fig. S13B, C). In CFA model animals exhibiting comorbidity of pain and anxiety behaviors, we next used a genetically encoded GABA sensor (AAV2/9-DIO-iGABASnFR) to determine the dynamics of GABA release in and around the LH from LS projection axon terminals (Fig. S13, D). There were large increases in GABA release around the LH from LS during von Frey stimulation, center of OFT and open-arm exploration of EPM (Fig. S13E–G). These findings represent strong evidence that the LS^GABAergic^-LH circuit exhibits robust activation during exposure to nocifensive stimulation and it constitutes a key part of the neuronal substrate that functionally mediates anxiety-like behavior in CFA mice.

## Discussion

Chronic pain and psychiatric disorders, such as depression and anxiety, are frequently encountered together, each rendering the patient’s satisfactory recovery of the other condition mutually more difficult [21, 22], even though most findings have detected a common neuronal basis for these pathological states [5]. Addressing this context, we report several major findings: (i) chronic pain and anxiety interacted with each other, and pain threshold was positively correlated with the time in the center and open arms in mice; (ii) both chronic pain and anxiety increased excitability of LS GABAergic neurons; (iii) optogenetic/chemogenetic activation of LS GABAergic neurons lowered the pain threshold and induced anxiety-like behavior in naïve mice, and pain and anxiety shared the same cellular subsets regulated in LS; (iv) optogenetic/chemogenetic inhibition of LS GABAergic neurons ameliorated hyperalgesia and anxiety-like behavior in a mouse model of CFA or CRS; (v) CFA or CRS increased inhibitory synaptic input to LH from LS GABAergic neurons; and (vi) chemogenetic activation of LH-projecting LS not only decreased pain threshold but also induced anxiety-like behavior in naïve mice. Together, these results indicate that the activation of LS GABAergic or LH-projecting LS neurons plays a critical role in the comorbidity of pain and anxiety (Fig. S14).

Clinical studies have found that pain and anxiety may interact with each other, and even aggravate each other [5]. In this study, we did find an association between pain and anxiety: in chronic inflammatory pain, anxiety correlated with lowered pain threshold in mice. A similar correlation was found in the CRS model, although the change in the pain threshold there was not as pronounced as in CFA mice. In view of this finding, we propose that pain perception and emotional anxiety are probably processed in part by shared brain networks. The c-Fos protein, expressed by an immediate early gene, is generally used in neurons as a marker for firing activation [23]. Using whole-brain c-Fos staining, we demonstrated that both CFA and CRS activated LS neurons, a necessary condition for LS GABAergic neurons to play a role in the regulation of the comorbidity of pain and anxiety, including in CFA and CRS models. Moreover, with use of the TetTag-hM3Dq system, we were able to verify the active involvement of pain- and anxiety-responsive LS neurons in the modulation of comorbid pain and anxiety behaviors.

Under physiological conditions, the LS receives topographically organized inputs from the cortex and HPC and maintains appropriate excitability. LS is reciprocally connected with thalamic and midbrain structures and regulates appropriate emotional expression [24–26]. However, our fiber photometry recording showed that GABAergic neurons in LS were abnormally hyperactivated in pathological conditions, and the enhanced inhibitory LS output to downstream brain regions lead to the development of pain and anxiety. In fact, the activation/inhibition of GABAergic neurons in LS by using multiple manipulation methods resulted in enhanced/diminished occurrence of pain and anxiety comorbidities.

Other studies, however, have implied an anxiogenic role for the LS. One study found that activation of the type 2 CRF receptor (Crfr2) marks a subset of LS GABAergic neurons, which connected with the anterior hypothalamus and enhanced stress-induced anxiety-like behavior [19]. In addition, oxytocin receptor (OXTr) expression in a subset of LS neurons also exerted anxiogenic effects, mainly by inhibiting HDB neurons via inhibitory GABAergic projections to the HDB [27]. Our study is consistent with these findings but goes further by refining the previous observations with the newly revealed role of LS to HDB projection in the regulation of anxiety, but not in pain regulation. More importantly, we also revealed that chemogenetic activation of LH-projecting LS neurons facilitated the mechanical pain threshold and induced anxiety-like behavior in naïve mice. Additionally, fiber photometry recordings indicated that in animals exposed to pain and anxiety-provoking environments, such as the von Frey stimuli, EPM, or OFT, LH-projecting LS neurons, overall, represent pain and anxiety-related features, and this representation is used by the animal to guide pain and anxiety-related behavior. The PAG is another well-validated site for analgesia [28, 29]. In this study, we traced LS GABAergic projections to the dorsolateral part of the PAG and found that the functionally activated LS^GABAergic^-PAG circuit induced pain, but no anxiety. In other words, distinct LS populations engaged differently in the regulation of pain and anxiety at the circuit level. These findings suggest that one or the other distinct LS GABAergic neuron subpopulation and its respective projection targets in other brain regions, such as the LH, HDB and PAG, may be the neuronal mechanism underlying some form of sensory or emotional dysfunctions.

In fact, we used retrograde tracing technology to show that distinct sets of LS neurons projected to HDB, LH or PAG. Traditionally, the LS has been divided into three main divisions based on their distinct major afferents and efferents. Our anterograde tracing experiment also revealed differences between dorsal and ventral LS projections to downstream brain regions. Different LS projections determined their functional difference, we only focused on the overall role of LS GABAergic neurons in pain and anxiety comorbidities. The differences in function and network connections between LS subregions associated with distinct mechanisms of pain and anxiety processing need to be further explored in the future.

According to an early theory, the LS has an important part in the septohippocampal system. This theory conceptualized the system as a network of emotion and affect processing nodes [19, 30–32]. Past studies have focused on the role of vHPC projections to LS in the regulation of anxiety [33]. However, to explore the function of LS projections to the ventral HPC, we failed to find a wanted effect on anxious and pain behavior. It is possible that the projection from hippocampus to LS is involved in the regulation of anxiety, and conversely, the projection from LS to hippocampus has no regulatory effect on anxiety, or there are differences in the participation of the projection from LS subregions to hippocampus in the regulation of anxiety. In this study, the participation of the projection from whole LS in the regulation of anxiety will eventually counteract the effect of each other. Differences in LS subregion projection to the downstream, and its role in the regulation of pain and anxiety will be the subject of our study. Manipulation of the other two pathways, LS-MPOA and LS-VTA circuits had no effect on both anxiety and pain.

In classical lesions of the septum, including both its medial and lateral subdivisions, animals exhibit “septal rage,” marked by increased aggressiveness, hyperactivity, and hyperdefensive behaviors [34]. Reversible inactivation of the LS yielded similar phenotypes [35, 36]. These and other data have led to a prevailing view that LS output is anxiolytic, i.e., dampens fear or anxiety. However, other studies have implied an anxiogenic role for the LS [6]. This study did not distinguish the subregions of LS, and studied the role of overall LS in pain and anxiety, and LS activation induced the occurrence of pain and anxiety comorbidities. It remains to be determined whether a persistent anxiolytic pathways exists or if persistence is rather a unique property of circuits that elevate anxiety.

In conclusion, our findings reveal the critical role of LS GABAergic neurons in the regulation of pain and anxiety comorbidities, and establish the role of GABAergic LS-to-HDB, LH-to-PAG, and LS-to- LH projections in the regulation of anxiety, pain, and both, respectively. Furthermore, strategies targeting neuronal circuits that include LH-projecting LS GABAergic neurons have therapeutic potential for pain and anxiety disorders.

## Supplementary information

Supplementary Material

Table S1

## Data Availability

All data needed to evaluate the conclusions in the paper are present in the paper or the supplementary materials.
