# Supplementary material for: Lateral septum-lateral hypothalamus circuit dysfunction in comorbid pain and anxiety
Source: Mol Psychiatry. 2023 Jan 16;28(3):1090–100. doi: 10.1038/s41380-022-01922-y (PMC10005966; doi:10.1038/s41380-022-01922-y)
Supplement: Supplementary file 1 — Supplementary Material [file 41380_2022_1922_MOESM1_ESM.docx]

*Supplementary Material*

**Title:**

**Lateral septum-lateral hypothalamus circuit dysfunction in comorbid pain and anxiety**

**Authors:**

Di Wang^1†^, Xiangyu Pan^2†^, Yu Zhou^2†^, Zifeng Wu^1†^, Kunpeng Ren^2^, Hanyu Liu^1^, Chaoli Huang^1^, Yumei Yu^2^, Teng He^1^, Xiao Zhang^3^, Ling Yang^4^, Hongxing Zhang^2^, Ming-Hu Han^5^, Cunming Liu^1*^, Jun-Li Cao^2*^, Chun Yang^1*^

**Affiliations:**

^1^Department of Anesthesiology and Perioperative Medicine, The First Affiliated Hospital of Nanjing Medical University; Nanjing, China;

^2^Jiangsu Province Key Laboratory of Anesthesiology, Jiangsu Province Key Laboratory of Anesthesia and Analgesia Application Technology, Xuzhou Medical University; Xuzhou, China;

^3^Department of Anesthesiology, The Affiliated Wuxi NO. 2 People's Hospital of Nanjing Medical University; Wuxi, China;

^4^Department of Cardiology, The Third Affiliated Hospital of Soochow University; Changzhou, China;

^5^Department of Mental Health and Public Health, Faculty of Life and Health Sciences, Shenzhen Institute of Advanced Technology, Chinese Academy of Sciences; Shenzhen, China.

***Corresponding author:**

Chun Yang, Email: [chunyang@njmu.edu.cn](mailto:chunyang@njmu.edu.cn).

Jun-Li Cao, Email: [caojl0310@aliyun.com](mailto:caojl0310@aliyun.com).

Cunming Liu, Email: [cunmingliu@njmu.edu.cn](mailto:cunmingliu@njmu.edu.cn).

†These authors contributed equally to this work.

**List of Supplementary Materials**

Present a list of the Supplementary Materials in the following format.

Materials and Methods

Fig. S1 to Fig S14 for multiple supplementary figures

Table S1 for Extended statistical information

**Contents**

1. Abbreviations
2. Methods
   1. Animals
   2. Chronic pain model: inflammatory pain induced by complete Freund’s adjuvant (CFA)
   3. Chronic anxiety model: anxiety induced by chronic restraint stress (CRS)
   4. Stereotaxic surgery and injection sites
   5. Viral injections
   6. Mechanical hyperalgesia–von Frey pain threshold measurement
   7. Open field test (OFT)
   8. Elevated plus maze (EPM) test
   9. Tail suspension test (TST)
   10. Forced swim test (FST)
   11. Fiber photometry
   12. Fiber photometry data analysis
   13. Genetic lesion experiments
   14. Chemogenetic protocols
   15. In vivo optogenetic manipulation
   16. TetTag-chemogenetic behavioral protocols
   17. In vivo electrophysiology recordings
   18. Photogenetic and chemogenetic verification experiments
   19. Analysis of single unit spike responses
   20. Immunohistochemistry
   21. Quality control
   22. Statistical analysis
3. Supplementary Figures

Fig. S1 - Chronic pain and chronic stress both fail to induce depression-like behaviors

Fig. S2 - Chronic pain and chronic stress leads to a decrease of body weight in mice

Fig. S3 - GABAergic neurons in LS are activated by chronic pain and anxiety

Fig. S4-Serial re-activation of genetically tagged neuronal ensembles following acute pain stimulation or acute restraint stress

Fig. S5 - Genetic ablation of LS GABAergic neurons attenuates pain aversion and anxiety behaviors

Fig. S6 - Chemogenetic inhibition of LS GABAergic neurons promotes resilience to comorbidity of pain and anxiety

Fig. S7 - Tracing LS GABAergic neurons pathway with H129-ΔTK-tdT

Fig. S8 - Distinct patterns of brain-wide efferent projections from dorsal and ventral LS

Fig. S9 - Effects of chemogenetic activation of LS-HDB, LS-LH, and LS-PAG circuits on behavioral effects

Fig. S10 - Effects of chemogenetic activation of LS-mPOA, LS-vHPC, and LS-VTA circuits on behavioral effects

Fig. S11 - LS neurons that project to HDB, LH, and PAG are distinct subpopulations

Fig. S12-Nocifensive and anxiety-like behaviors are associated with the dynamics of LS-HDB, LS-LH and LS-PAG neurons.

Fig. S13 - Dissection of the LS→LH circuit.

Fig. S14-The role of LS and its downstream projection pathway regulating hyperalgesia and anxiety behaviors.

1. References

**1. Abbreviations**

ARC, arcuate hypothalamic nucleus

CFA, complete Freund’s adjuvant

CRS, chronic restraint stress

DM, dorsomedial hypothalamus

FST, forced swim test

HDB, horizontal limb of the diagonal band

HPC, hippocampus

LH, lateral hypothalamus

LPO, lateral preoptic area

LS, lateral septum

MPOA, medial preoptic area

PAG, periaqueductal gray

SNC, substantia nigra pars compacta

TST, tail suspension test

VDB, vertical limb of the diagonal band

vHPC, ventral hippocampus

VLPO, ventrolateral preoptic area

VMH, ventromedial hypothalamus

VTA, ventral tegmental area

**2. Materials and Methods**

**2.1** **Animals.** Male C57BL/6 mice (Beijing Charles River Laboratory Animal Breeding Co. Ltd., China) and female and male *Vgat-ires-Cre* transgenic mice (*Vgat-ires-Cre:* Slc32a1tm2(Cre)Lowl/J, RRID: 016962; The Jackson Laboratory, ME, USA) were purchased at age 8–12 weeks and housed 4–5 per cage. The animals were maintained at a controlled temperature (24 °C ± 2 °C) with 12:12 h light/dark cycle (lights on at 07:00) and ad libitum access to food and water. Only adult male mice were used in the behavioral experiments. Adult male and female mice with a similar distribution were used for adeno-associated virus (AAV) and herpes simplex virus (H129) anterograde tracing and in vivo electrophysiological experiments.

All procedures were approved by the Institutional Animal Care and Use Committee of Nanjing Medical University and Xuzhou Medical University and were performed in accordance with the National Institutes of Health Guide for the Care and Use of Laboratory Animals.

**2.2 Chronic pain model: inflammatory pain induced by complete Freund’s adjuvant** **(CFA).** Chronic inflammatory pain was induced in mice by subcutaneously injected 10 μl complete Freund’s adjuvant (CFA, 1 mg/ml heat-killed Mycobacterium tuberculosisin dissolved in 85% paraffin oil and 15% mannide monoleate) (Abcam, USA) (*37, 38*) into the planta of left hind paw after the skin was disinfected with iodine tincture. Saline (0.9% NaCl) was similarly volume injected as the control.

**2.3 Chronic anxiety model: anxiety induced by chronic restraint stress (CRS).** Mice were physically restrained in 50 ml falcon tubes for 2 h (between 10:00 a.m. and 12:00 a.m.) daily for 7 consecutive days. The tubes were well-ventilated through holes drilled in the ends. During the restraint period, the animals in the control group were allowed to freely move in their home cage under normal conditions but without access to water or food during. The tubes were thoroughly washed after the restraint every day (*39*). To preclude confounds from acute stress, the mice were allowed to rest 1 day after CRS before the anatomical and behavior experiments.

**2.4 Stereotaxic surgery and injection sites.** *Vgat-Cre* mice were anesthetized using intraperitoneally (i.p.) injected sodium pentobarbital (50 mg/kg) before they were placed in a stereotaxic apparatus (Shanghai Yuyan Instruments Co., Ltd., China). Ophthalmic ointment was administered to prevent drying of the eyes. The animals were lying on a heating pad to maintain the body temperature at 35 °C to 37 °C. To prepare the incision site for cranial surgery, scalp hair was shaved oﬀ and the skin was disinfected using iodine and medical alcohol. The scalp was incised to expose the skull, and the connective tissue adhering to the skull was gently removed using cotton swabs. Craniotomy holes of ~1 mm diameter was drilled under a surgical microscope.

The stereotaxic coordinates were defined as dorsal-ventral (DV) from the brain surface, anterior--posterior (AP) from bregma and medio-lateral (ML) from the midline (in mm). The brain regions and the coordinates (AP, ML, DV) of the injection targets were as follows: LS (+0.80, ±0.40, −3.50), dorsal LS (+0.80, +0.40, −3.00), ventral LS (+0.80, +0.40, −3.80), HDB (+0.74, ±0.80, −5.50), LH (−1.58, ±1.00, −5.00), PAG (−3.50, ±0.40, −2.50), MPOA (−0.10, ±0.50, −5.00), vHPC (−2.90, ±2.20, −4.70), VTA (−3.20, ±0.50, −4.50).

**2.5 Viral injections.** Adeno-associated virus (AAV) vectors (all from BrainVTA, China; see other specifics below) were injected (100~200 nl) at a rate of 0.1 µl/min through a stainless steel 33-gauge/15-mm/PST3 internal cannula attached to a 10-µl syringe (Hamilton, USA). After injection, the pipette was left in place for an additional 10 min and then slowly retracted.

For the fiber photometry experiments, we injected the unilateral LS of *Vgat-Cre* mice with recombinant AAV2/9-DIO-GCaMP6s (titer: 5.56×10^12^ vg/mL) or control AAV2/9-DIO-EGFP (titer: 5.40×10^12^ vg/mL). The C57BL/6 mice were unilaterally injected with AAV2/1-Cre (titer: 1.00×1013 vg/mL) into the LS and AAV2/9-DIO-GCaMP6s into the HDB, LH or PAG.

For the detection the GABA release from LS to LH in C57BL/6 mice, we injected the unilateral LS with AAV2/1-Cre (titer: 1.00×10^13^ vg/mL), which enabled the Cre enzyme to spread anterogradely and monosynaptically into the soma of projection target LH neurons. Then, the bilateral LH were injected with GABA sensor AAV2/9-DIO-iGABASnFR (titer: 2.87×10^12^ vg/mL).

For the selective ablation of LS GABAergic neurons, we bilaterally injected the LS with AAV2/9-DIO-taCasp3-mCherry (titer: 2.97×10^12^ vg/mL) or AAV2/9-DIO-mCherry (titer: 5.14×1012 vg/mL) in *Vgat-Cre* mice.

Additionally, for the experiments of selective optical activation or inhibition of LS GABAergic neurons, we bilaterally injected the LS with AAV2/9-DIO-ChR2-mCherry (titer: 5.00×10^12^ vg/mL), AAV2/9-DIO-mCherry (titer: 5.14×10^12^ vg/mL), or AAV2/9-DIO-NpHR-mCherry (titer: 5.24×10^12^ vg/mL) in *Vgat-Cre* mice.

For the chemogenetic activation or inhibition of LS GABAergic neurons in *Vgat-Cre* mice, we bilaterally injected the LS with AAV2/9-DIO-hM3Dq-mCherry (titer: 5.27×10^12^ vg/mL), AAV2/9-DIO-mCherry (titer: 5.14×10^12^ vg/mL), or AAV2/9-DIO-hM4Di-mCherry (titer: 5.18×10^12^ vg/mL). For the LS injections of TetTag-DREADD, the two tag-associated AAVs (AAV-PcFos-tTA and AAV-PTRE-tight-hM3Dq-mCherry; titer: 5.23×10^12^ vg/mL) were premixed in an equal ratio.

The distribution pattern of collateral projection fibers from LS GABAergic neurons, we injected the unilateral LS with AAV2/9-DIO-EGFP (titer: 5.40×10^12^ vg/mL) in *Vgat-Cre* mice. Distribution pattern of projection fibers of GABAergic neurons in different subregions of LS, we injected the unilateral dorsal LS with AAV2/9-DIO-EGFP (titer: 5.40×10^12^ vg/mL) and the ventral LS with AAV2/9-DIO-mCherry (titer: 5.14×10^12^ vg/mL) in *Vgat-Cre* mice.

For the monosynaptic anterograde tracing of LS projections in *Vgat-Cre* mice, Then, to express thymidine kinase (TK) in *Vgat-Cre* cells, the same unilateral LS sites of the same animals were injected with AAV2/9-DIO-TK virus (helper virus) (titer: 3.62×10^12^ vg/mL). Then, 21 days after the first injection We injected a high titer (titer: 1.50×10^9^ vg/mL) of a genetically modified version of Herpes simplex virus type 1 strain 129 (H129-ΔTK-tdT virus) at the same injection site as above. Five days later, mice were killed for analysis and brains were fixed. Twenty-four hours after fixation, brain sections were imaged under a laser confocal microscope (Zeiss LSM 880, Carl Zeiss, Germany). With the assistance of the helper virus, H129-ΔTK-tdT is anterogradely transmitted through *Vgat-Cre* cell synapses and labels postsynaptic neurons as previously described.

For the monosynaptic retrograde tracing experiments, we unilaterally injected the HDB with retro-AAV-hSyn-mCherry (titer: 5.13×10^12^ vg/mL), the LH with retro-AAV-hSyn-EGFP (titer: 5.65×10^12^ vg/mL), and the PAG with retro-AAV-hSyn-mTagBFP-3XFlag (titer: 5.07×10^12^ vg/mL). For monosynaptic antegrade neural circuit manipulation, we injected the bilateral LS of C57BL/6 mice with AAV2/1-Cre (titer: 1.00×10^13^ vg/mL), which enabled the Cre enzyme to spread anterogradely and monosynaptically into the soma of LS projection target neurons of HDB, LH, and PAG neurons. Then, we bilaterally injected into HDB, LH, and PAG Cre-dependent AAV2/9-DIO-hM4Di-mCherry (titer: 5.18×10^12^ vg/mL) to enable the chemogenetic manipulations of the LS-HDB, LS-LH, and LS-PAG circuits.

For retrograde tracing of LS projections to the HDB, LH, PAG, MPOA, vHPC, and VTA, we injected retro-AAV-Cre virus (titer: 6.24 × 10^12^ vg/mL) into the bilateral HDB, LH, PAG, MPOA, vHPC, and VTA of C57BL/6 mice. For the manipulation of specific neurons in the LS, we injected the bilateral LS with AAV2/9-DIO-hM3Dq-mCherry (titer: 5.27×10^12^ vg/mL).

**2.6 Mechanical hyperalgesia–von Frey pain threshold measurement.** Before the test, each mouse was placed in a plastic box with a mesh floor and allowed to acclimate for 1 h. A series of calibrated von Frey filaments (with force markings of 0.008, 0.02, 0.07, 0.16, 0.4, 1.0, 2.0, and 6.0 g) were perpendicularly applied in increasing order to the plantar surface of the hind paw with sufficient force to bend the filaments. Brisk hind paw movements (withdrawal or flinching) were taken as a positive pain response. Lifting of the paw as part of normal locomotor behavior was ignored. In the absence of a paw withdrawal response to the selected hair, a filament of the next greater force was applied. In the event of paw withdrawal, the next weaker stimulus was chosen. We defined pain threshold, $50\% g threshold$, as the tactile stimulus producing a 50% likelihood of withdrawal, we used Dixon’s up-down method to determine it (*40*) . According to the Dixon method, pain threshold calculation requires 6 responses in the immediate vicinity of the 50% threshold. Then $\mathrm{threshold}_{pain,50\%}$ was obtained by interpolated using the formula below:

50% g threshold = (10^[Xf+k𝞭]^)/ 10,000

where, X_f_ is the force (in log units) of the final von Frey hair used; k is Dixon’s tabulated value for the observed succession pattern of positive/negative responses; and δ is mean difference (in log units) between stimuli in the force series. The baseline $50\% g threshold$ was measured before optical stimulation.

For optical stimulation of the LS, we followed the stimulation protocol described in earlier study (*41*) with 473 nm blue light and 589 nm yellow light delivered directly to the LS for 15 min. All tests were conducted blind to genotype or treatment group (mice were randomly assigned to different groups), and the experimenter (who was blind to the wavelength of the light stimulation used).

**2.7 Open field test (OFT).** The OFT was used to evaluate locomotor activity of mice. Mice were placed in the center of a polystyrene enclosure (40 cm × 40 cm × 35 cm) and allowed to move freely for 5 min and were videotaped individually. The “center area” was defined as the 20 cm × 20 cm rectangular area centered on the midpoint of the field. The open field was cleaned with 75% ethanol between each trial. The video recordings of the animal’s track were analyzed offline using the YHOFData software (Wanhan Yihong Technology Co., Ltd, China), extracting total distance traversed (m) as a measure of locomotor activity and time spent in the center area as an (inverse) measure of anxiety-related behavioral measures.

**2.8 Elevated plus maze (EPM) test.** The EPM consisted of two risk-laden arms (each with a 30 cm × 5 cm open area without sidewalls) and two “safe” arms (each with a 30 cm × 5 cm area enclosed by 20 cm high walls on the sides and the end), connected with a central platform (5 cm × 5 cm) elevated 40 cm above the floor. Mice were placed in the central platform such that they faced an open arm and were allowed to explore freely for 5 min. Animal behaviors were captured on video recordings and analyzed by using the EPMdata software (Wanhan Yihong Technology Co., Ltd, China). Time spent in the open arms was extracted as a measure of the anxiety levels of mice. The total number entries made into the 4 arms was extracted as a measure of the animal’s locomotor activity.

**2.9 Tail suspension test (TST).** Mice were individually suspended by their tails with tape in the rectangular compartment (82 cm long × 27 cm width × 80 cm height) of a specially manufactured two-walled tail suspension box (25 cm long × 27 cm width × 80 cm height). Each mouse was only tested once, for 6 min, and the test was videotaped from the side. The immobility time of the animal was extracted from the last 5 min of the recording by using the YHTSData software (Wanhan Yihong Technology Co., Ltd, China).

**2.10 Forced swim test (FST).** The mouse was placed in a transparent Plexiglas cylinder (25 cm height x 16 cm diam) that filled with fresh water (24 °C ± 1 °C) up to a height of 20 cm from the bottom and left to swim for 6 min. The behavior of the mouse was videotaped from the side. The total duration of immobility during the last 5 min was extracted offline by using the YHTSData software (Wanhan Yihong Technology Co., Ltd, China). Mice from the control and experimental groups were tested by turns. A mouse was considered immobile when remaining motionless or floating.

**2.11 Fiber photometry.** We recorded localized calcium signals gauged by fiberoptic photometry of GCaMP6s fluorescence. Three days before the behavioral tests and local fiber photometry recordings, mice were injected with GCaMP6s before unilaterally implanted with optical fibers (200 𝑢m O.D., 0.37 NA, Inper, Nanjing, China) in the LS. The fiber photometry system (ThinkerTech Nanjing Bioscience Inc., China) has been described previously (*42*). Briefly, For GCaMP6s excitation, a 488-nm laser beam (OBIS 488LS, Coherent) was reflected off a dichroic mirror (MD498, Thorlabs), focused with a 10× objective lens (0.3 NA, Olympus Inc., Japan), and then coupled to an optical commutator (Doris Lenses). The commutator and implanted fiber were connected by a 2-m optical fiber (200 𝑢m O.D., 0.37 NA). The laser power at the tip of the optical fiber was adjusted to 20–40 μW. The GCaMP6s fluorescence signal was bandpass filtered (MF525-39, center wavelength, 525 nm, bandwidth, 39 nm, Thorlabs). The photomultiplier tube current output was amplified and converted into a voltage signal by using an amplifier, then digitized at 50 Hz and recorded using a Power 1401 digitizer with the Spike2 software (CED, Cambridge, UK). Heatmaps and averaged Ca^2+^ traces were plotted using custom-written functions in MATLAB (version 2017b, The Math Works, Inc., USA).

**2.12 Fiber photometry data analysis.** Raw photometry data (filtered and digitized voltage signals) were exported to MATLAB for further analysis. The raw photometry signal was converted to ΔF/F relative variations, i.e., ΔF = F–F_0_ deviations relative to the F_0_ baseline fluorescence signal. To analyze neuronal activity time-locked to behavioral activity, we determined the time point of transition for each behavioral transition and windowed the ΔF/F time series within a ±10 s window centered on the time point of transition. This approach was applied to the analysis of data before-after von Frey stimulation, and transitions from EPM center to open arms, and from OFT-around to OFT-center. We checked and verified that the photometry signals were free of movement artifacts, as the GFP signal originating from control AAV2/9-DIO-GFP expression in LS GABAergic neurons did not change during epochs of anxiety behavior.

**2.13 Genetic lesion experiments.** To lesion LS GABAergic neurons, AAV2/9-DIO-taCasp3-mCherry was injected bilaterally into the LS of *Vgat-Cre* mice. In the control group, the same volume of AAV2/9-DIO-mCherry was injected bilaterally into the LS of *Vgat-Cre* mice. Pain thresholds and OFT and EPM behaviors were measured at post-injection days 3, 7, 14, and 21 for CFA mice and post-injection day 21 for CRS mice.

**2.14 Chemogenetic protocols.** Mice were randomized to receive [intraperitoneal](http://www.baidu.com/link?url=Y1jmoVR_lbFBlFYvTCW5nGFyZhSmWnfXupT578MPJwOxhToHyfAfJFJ0NuNSTl64_dxpmgQm14hytN1hC3--fbtg3k9Y60WvraG53dRhihNWIi8sjyhaMwNUAX0QWXJK)ly injected 2 mg/kg clozapine-N-oxide (CNO) (HY-17366, MedChen Expression, USA) or saline of the equivalent volume. To determine the eﬀects of chemogenetic activation or inhibition of LS GABAergic neurons, pain thresholds were measured at post-injection 0.5 h, 1.5 h, 2.5 h, and 4 h. Anxiety-like behaviors were only monitored and evaluated at 30 min after injection.

**2.15 In vivo optogenetic manipulation**. In these experiments, the behavior of mice was measured in the hyperalgesia, OFT, and EPM experiments, while the activity of LS GABAergic neurons was optogenetically manipulated by continuous pulse illumination (2–3 mW, 5 ms pulse, 20 Hz) in the bilateral LS. LS GABAergic neurons were activated by blue (473 nm) light and inhibited by yellow light (598 nm). Behavioral assays were performed during light stimulation. The location of the fibers was examined after all the experiments.

**2.16 TetTag-chemogenetic behavioral protocols.** After AAV injection to create TetTag-DREADD mice, mice were raised on doxycycline treated (200 mg/L) drinking water for 28 days (*42*). Behavior conditioning experiments were done 2 days after the withdrawal of doxycycline. In experiments assessing anxiety behaviors induced by pain-related neurons, the mice received hind paw pain stimulation by applying the 1.0 g von Frey filament every 5 min over a 1.5 h test period. In experiments assessing hyperalgesia induced by anxiety-related neurons, the mice were subjected to 2 h acute restraint stress. Mice in both states were then returned to the doxycycline treatment (200 mg/L) through their drinking water for the next 4 days. Then behavior assessment experiments were conducted. CNO (2 mg/kg, i.p.) or saline was injected into the TetTag-DREADD mice, and 30 min post-injection the mechanical pain threshold and anxiety behaviors were tested in animals.

**2.17 In vivo electrophysiology recordings.** Adult *Vgat-Cre* mice were injected (200 µl/site) with recombinant AAV vectors (AAV2/9-DIO-ChR2-eYFP, AAV2/9-DIONpHR-eYFP, AAV2/9-DIO-hM3Dq-mCherry, or AAV2/9-DIO-hM4Di-mCherry) into the LS bilaterally as described above. Recordings were performed at 3 weeks post-injection or later to allow for label expression. The mice were anesthetized using 2% isoflurane and then maintained at 1.5% isoflurane on a stereotaxic apparatus, while body temperature was maintained at 35–37 °C using a heating pad. A single-channel optrode was constructed by assembling an optic fiber (0.48 NA; ThinkerTech Nanjing Bioscience) parallel to a glass-coated tungsten electrode (1–2 MΩ; Alpha Omega Engineering, Israel). The electrode was lower than the fiber by a vertical offset of ~100–200 μm between the tips. A custom-made single-channel injection electrode was attached to the front of the electrode in parallel. (This was a medium filament drug delivery pipeline fabricated from a 200-µl pipette nozzle by burning and fusing under an alcohol lamp). The tip (drug delivery port) was approximately 100–200 µm from the tip of the electrode, and the input end was connected to a microsyringe. After collection of electrophysiological data, mice were deeply anesthetized with 1% sodium pentobarbital (50 mg/kg, i.p.) and underwent electrolytic lesion. The animals were sacrificed, and the brains were extracted and kept for histological analysis of electrode location.

**2.18 Photogenetic and chemogenetic verification experiments.** The skull window was opened at the top of the target brain region, and the position coordinates of the LS (AP: +0.8 mm, ML: ± 0.4 mm, DV: –3.0 to –3.8 mm) and LH (AP: −1.58 mm, ML: ± 1.0 mm, DV: −4.8 to −5.2 mm) were recorded. The spikes were amplified (20×) using a diﬀerential amplifier, digitized (at 44 kHz), and stored on the computer using recording software from AlphaOmega SnR (Israel). Once the spikes from visually responsive units were isolated, optical stimulation or CNO was administered for optogenetic or chemogenetic intervention to observe the eﬀect of the intervention on the firing of the isolated unit.

**2.19 Analysis of single unit spike responses.** Off-line analysis of the action potentials recorded with the extracellular electrode was performed using custom-written MATLAB functions. The spike waveforms were normalized to unit full (peak to trough) amplitude, and then subjected to principal component analysis. The first two principal components were extracted and used for a 2-dimensional cluster analysis of spike events to detect isolated units based on the similarity of spike waveforms (*K*-means sorting method) *(43,44)*. The spikes of a single unit thus isolated were assumed to be recorded from the same single neuron.

To assess how tweezers stimulation affected single unit responses, we compared the spike counts in a 2 s window both before and after the stimulation. Firing rates were calculated from the peri-stimulus time histogram using 50 ms bins. To detect light-induced changes in the firing rates recorded for *Vgat-Cre* GABAergic neurons activated or inhibited by the 20 Hz stimulation during the ‘light On’ epoch, we compared the spike counts in 10 s-long counting windows 5 s before and after the light onset. In the chemogenetic validation experiment, we compared the spike counts in 60 s-long counting windows 30 s before and after the CNO perfusion.

**2.20 Immunohistochemistry.** The mice were deeply anesthetized using 1% sodium pentobarbital (50 mg/kg, i.p.) and perfused transcardially with 20 ml of phosphate-buﬀered saline (PBS), followed by 20 ml of ice-cold 4% paraformaldehyde in PBS. The brains were carefully extracted from the skull and postfxed in 4% paraformaldehyde for 6 h. Next, fixed brains were dehydrated in a 30% sucrose bath at 4 °C until the brain sank to the bottom of the solution. Then 30-μm thick coronal sections were serially cut using a freezing microtome (CM 3050S, Leica Microsystems, Germany). Free-floating sections were washed 3 times in PBS for 10 min and blocked by incubation in phosphate buﬀered-saline with 1% bovine serum albumin (V900933-100G, Sigma–Aldrich, USA) and 0.1% Triton X-100 for 1h. Sections were incubated with primary antibody diluted in PBS and 0.1% Triton X-100 overnight at 4 °C on a shaker. The primary antibodies were rabbit anti–c-Fos (1:1,000; ABE457, Cell Signaling Technology, USA), mouse anti–GAD-67 (1:500; ab26116, Abcam, UK). (c-Fos is the protein product of immediate early gene expression that occurs in neurons following depolarizations. GAD-67, glutamic acid decarboxylase 67, synthesizes the inhibitory neurotransmitter GABA and can be used as a marker for the localization of GABAergic neurons.) Then slices were washed in PBS for 10 min 3 times and incubated in PBS for 2 h with a secondary antibody (Alexa Fluor 594 donkey anti-rabbit or Alexa Fluor 488 donkey anti-mouse, both 1:200; Invitrogen, USA). Finally, the samples were washed in PBS for 10 min at room temperature 4 times. Confocal images were acquired using the LSM 880 confocal microscope (Zeiss) and further processed using the microscope software ZEN (version 2.3, blue edition; Zeiss, Germany).

**2.21 Quality control.** All experiments and data analyses were conducted blindly, including, immunohistochemical, fiber photometry and behavioral analyses. The number of experimental replicates (n) indicated in the figure legends refers to the number of mice independently treated for each condition. We excluded animals in which optical fibers were found to be misplaced or virus transgene expression failed or expression was in the wrong locus.

**2.22** **Statistical analysis.** Prior to further analysis, each dataset was subjected to a normality test using the Shapiro–Wilk test. For datasets that were normally distributed, parametric tests of the variance and group differences (paired, unpaired *t*-tests, one-way or two-way ANOVA with LSD post hoc test) were used. Otherwise, the non-parametric Wilcoxon matched-pairs signed rank test, Mann Whitney Rank Sum Test, and the Kruskal Wallis One Way ANOVA on ranks with Uncorrected Dunn's test were used. For the analysis of correlations between the mechanical withdrawal threshold and OFT/EPM anxiety behaviors, we used the Pearson correlation for normally distributed data and the Spearman rank correlation otherwise. Statistical tests were performed using statistical software packages (MATLAB2017a, MathWorks.Inc, USA or GraphPad Prism 9, GraphPad Software.Inc, USA). Data are presented as the means ± SEM. Statistical significance was set at three levels (**p* < 0.05, ***p* < 0.01, ****p* < 0.001).

**
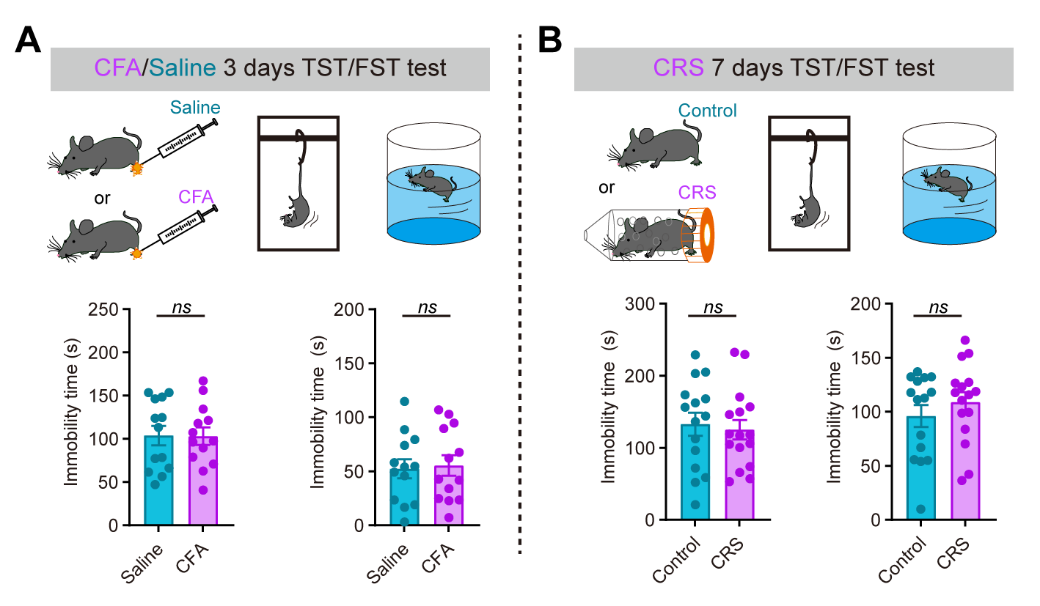
3. Supplementary Figures**

**Fig. S1. Chronic pain and chronic stress both fail to induce depression-like behaviors.** (**A**) Behaviors of mice treated with complete Freund’s adjuvant (CFA) to induce chronic pain or with saline (control) for 3 days in the tail suspension test (TST) and the forced swim test (FST). (**B**) Behaviors of mice subjected to chronic restraint stress **(**CRS) for 7 days and stress-naïve mice (control) in TST and FST. *ns*, no significant difference (*P* > 0.05). Error bars represent Data are presented as the means ± SEM. For further details of statistical data analysis see Table S1.


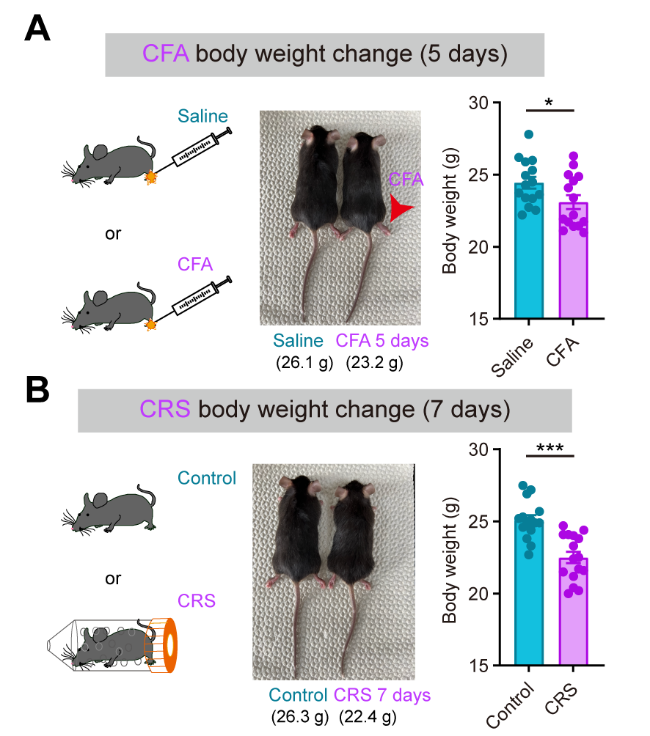
**Fig. S2. Chronic pain and chronic stress leads to a decrease of body weight in mice.** (**A**) Changes in body weight of mice treated with saline (control) or CFA for 5 days. (**B**) Changes in body weight of stress-naïve mice (control) and mice subjected to CRS for 7 days. **P* < 0.05, ****P* < 0.001. Data are presented as the means ± SEM. For further details of statistical data analysis see Table S1.


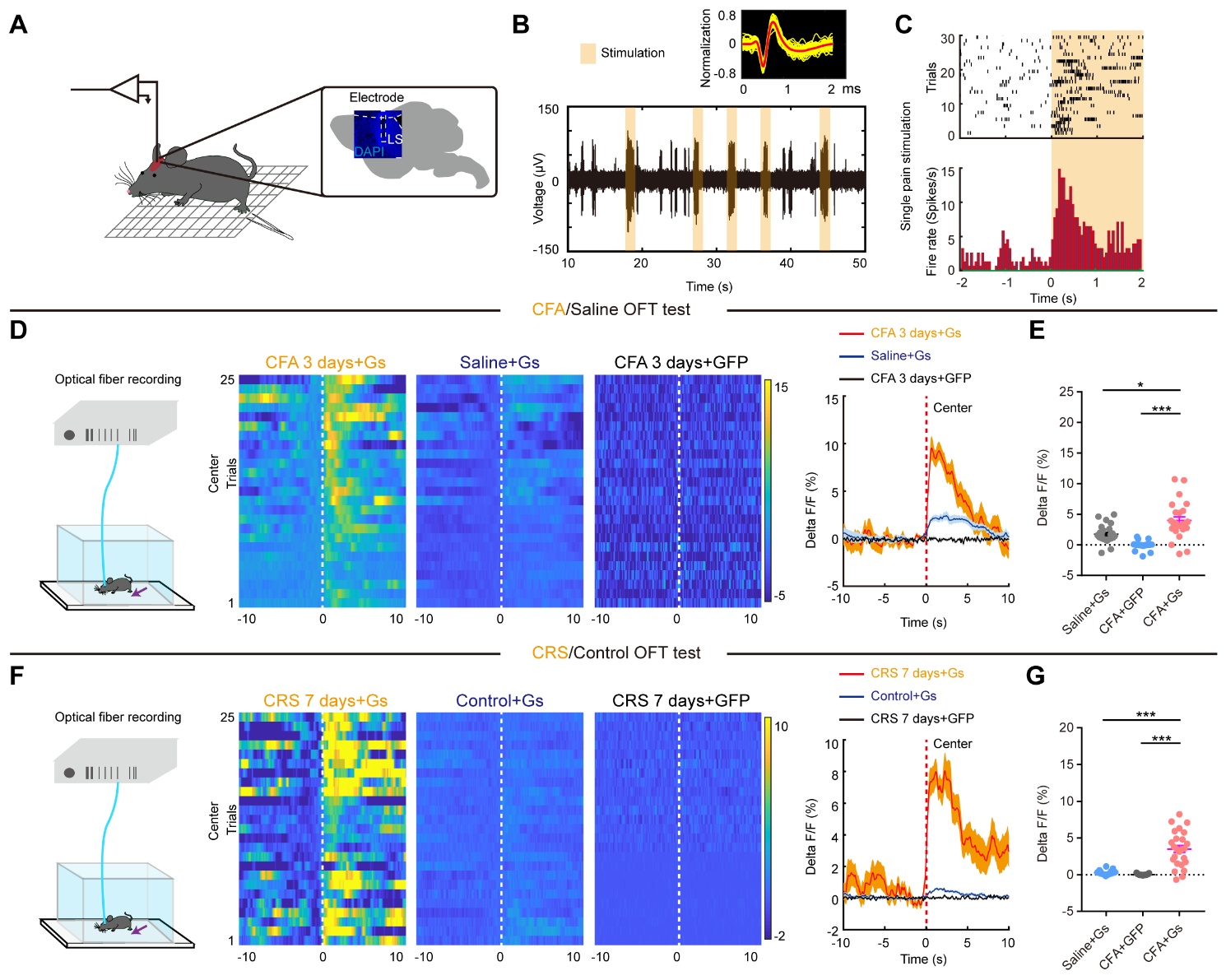
**Fig. S3. GABAergic neurons in LS are activated by chronic pain and anxiety.** (**A**) Single unit recording in the left LS while a light pain stimulus is delivered to the left paw with tweezers. A representative electrode tip position (electrolytically lesioned) is shown (right). Scale bar, 50 μm. (**B**) Normalized spike waves (top) and an example of single unit recording from the LS (bottom). The times of paw stimulation are indicated by the orange rectangles. (**C**) Raster plot of single unit firing and the resulting firing-rate histograms obtained following repeated trials of single-pulse optical stimulation in the LS. (**D**, **F**) Schematics of fiber photometry recording in freely moving mice. (Left) Animal entering in OFT center. (Middle) Trial-by-trial heatmap of Ca^2+^ transients recorded from LS GABAergic neurons before and after entry into OFT center. (Right) Temporal variation of the Ca^2+^ activity signal (ΔF/F) continuously recorded during the experiment and ΔF/F averaged for times before and during the procedure. (**E**, **G**) Comparison of the average over procedure ΔF/F recorded from LS GABAergic neurons in the saline/control or CFA/CRS groups within a 5s-window time-locked to the entry into the OFT center field. **P* < 0.05, ****P* < 0.001. Data are presented as the means ± SEM. For further details of statistical data analysis see Table S1.


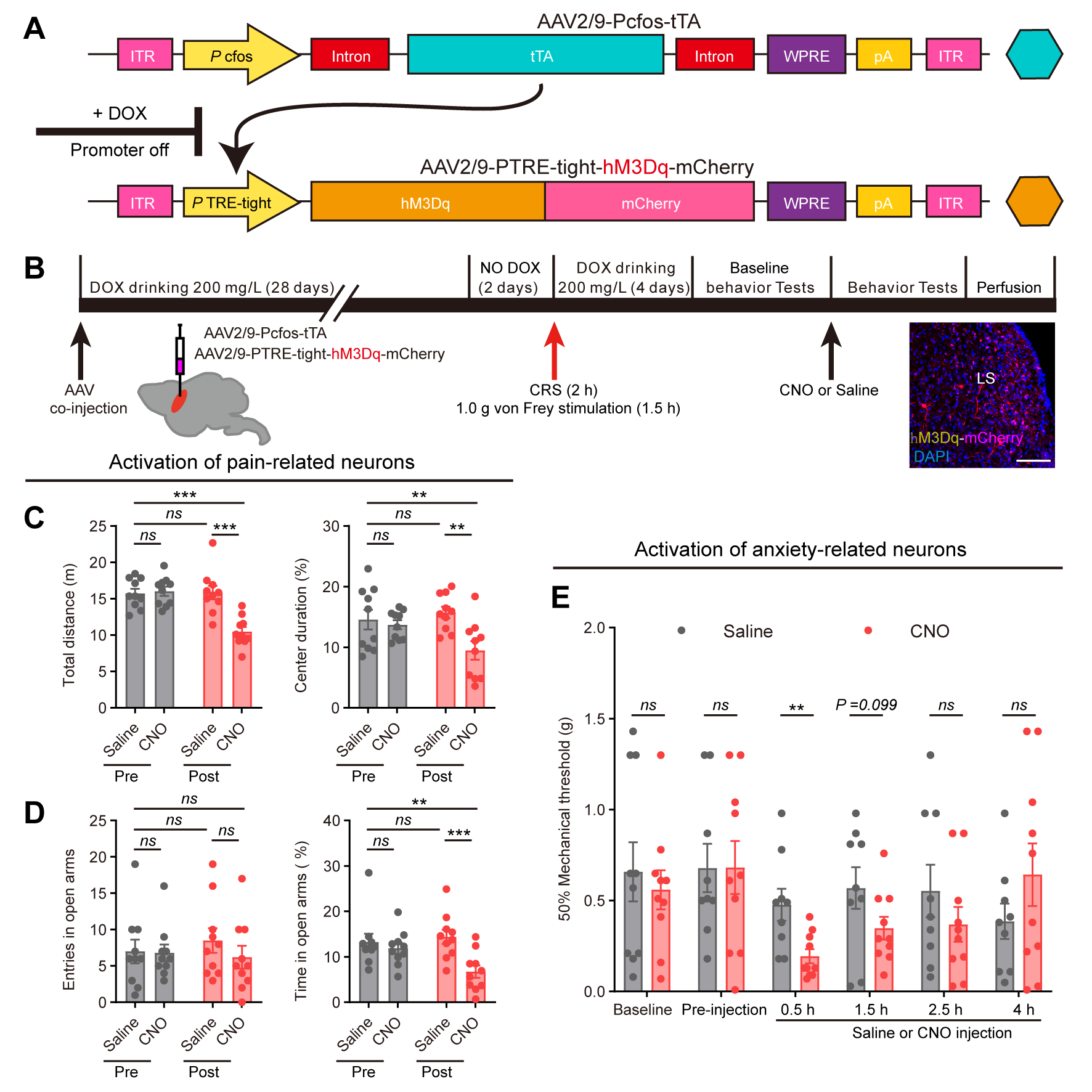


**Fig. S4. Serial re-activation of genetically tagged neuronal ensembles following acute pain stimulation or acute restraint stress**

(**A**) Two AAV transgenes were used, one with the c-Fos promoter, which drives in an activity dependent manner the expression of the tTA protein. In the presence of doxycycline (DOX), tTA cannot bind and activate its target promoter, PTRE-tight, coded in the second AAV vector. However, when doxycycline was removed, tTA activated hM3Dq-mCherry expression, but only in neurons in which tTA expression was driven by the c-Fos promoter, reflecting neural activity. (**B**) The experimental protocol and timeline, and a representative image of virus expression sites. Scale bar, 100 μm. (**C**) OFT results and (**D**) EPM test results before and after intraperitoneally injection of CNO re-activated LS pain-related neurons. (**E**) Mechanical pain sensitivity test before and after intraperitoneally injected CNO re-activated LS anxiety-related neurons. ***P* < 0.01, ****P* < 0.001. *ns*, no significant difference (*P* > 0.05). Data are presented as the means ± SEM. For further details of statistical data analysis see Table S1.DOX: doxycycline.


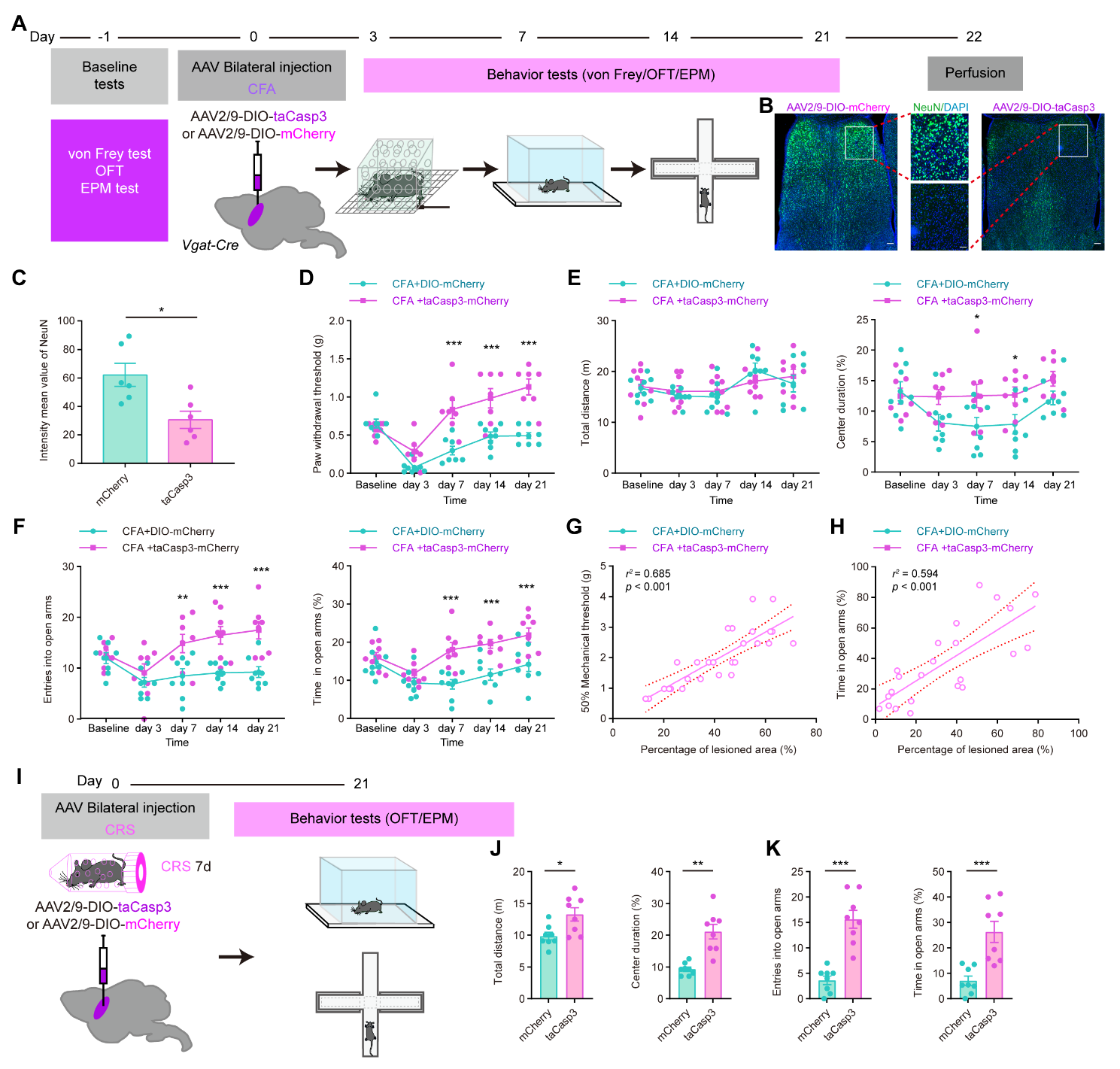
**Fig. S5. Genetic ablation of LS GABAergic neurons attenuates pain aversion and anxiety behaviors.** (**A**) Schematics of bilateral LS injection of taCasp3 to ablate GABAergic neurons; CFA modeling; and behavioral tests. (**B**) Neuron-specific nuclear (NeuN) protein staining from a control and an LS-lesioned mouse. The scale bar in LS represents 100 μm and 20 μm, respectively. (**C**) Intensity mean value of every section from NeuN-stained LS. (**D**) The mechanical pain thresholds observed over time after taCasp3 injection. (**E**) OFT at 3, 7, 14, and 21 days after taCasp3 injection. (Left) Percentage of time course of total distance. (Right) Percentage of time spent in the center. (**F**) EPM test at 3, 7, 14, and 21 days after microinjection with mCherry and taCasp3. (Left) Number entries into the open arms. (Right) Time spent in the open arms. (**G**, **H**) Correlation between the size of LS lesion (percentage LS area) and pain threshold (paw withdrawal threshold) or anxiety behavior (the time spent in open arms). (**I**) Experimental timeline, from LS lesioning to behavioral tests. (**J**) OFT results. (Left) Distance covered. (Right) Percentage of time spent in center. (**K**) EPM results 21 days after microinjection of taCasp3-mCherry or mCherry. (Left) Entries into arms. (Right) Percentage of time spent in open arms. **P* < 0.05; ***P* < 0.01; ****P* < 0.001. *ns*, no significant difference (*P* > 0.05). Data are presented as the means ± SEM For further details of statistical data analysis see Table S1.


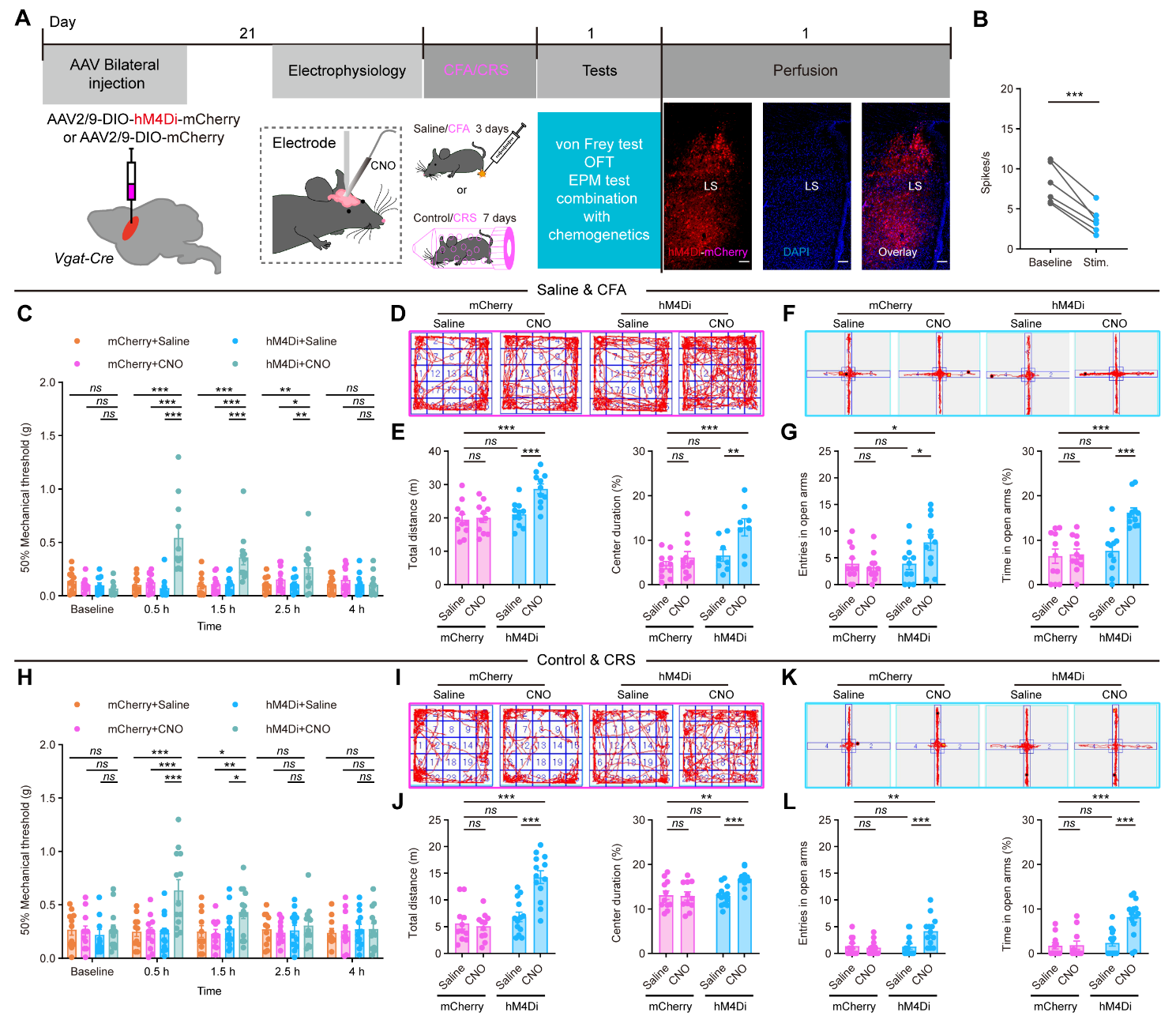
**Fig. S6. Chemogenetic inhibition of LS GABAergic neurons promotes resilience to comorbidity of pain and anxiety.** (**A**) Schematics of the experiment: virus injection, CFA and CRS modeling, and behavioral tests. Coronal sections featuring hM4Di-mCherry positive LS GABAergic neurons at the injection site. Scale bar, 100 μm. (**B**) The averaged the firing activity of LS GABAergic neurons upon CNO infusion. (**C**) The mechanical pain (paw withdrawal) threshold of CFA mice measured after saline or CNO injection, and repeated at 0.5 h, 1.5 h, 2.5 h, and 4 h after injection. (**D**) Representative exploration traces recorded for CFA mice in OFT. (**E**) Comparison of OFT results obtained for CFA animals in the mCherry versus hM4Di groups. (Left) Total distance covered. (Right) Percentage of time spent in the central area. (**F**) Representative exploration traces recorded for CFA mic in EPM. (**G**) Comparison of EPM results obtained for CFA animals in the mCherry versus hM4Di groups. (Left) Total entries into arms. (Right) Percentage of time spent in the open arms. (**H-L**) Same as (**C-G**) but obtained for CRS mice. **P* < 0.05, ***P* < 0.01, ****P* < 0.001. *ns*, no significant difference (*P* > 0.05). Data are presented as the means ± SEM. For further details of statistical data analysis see Table S1.


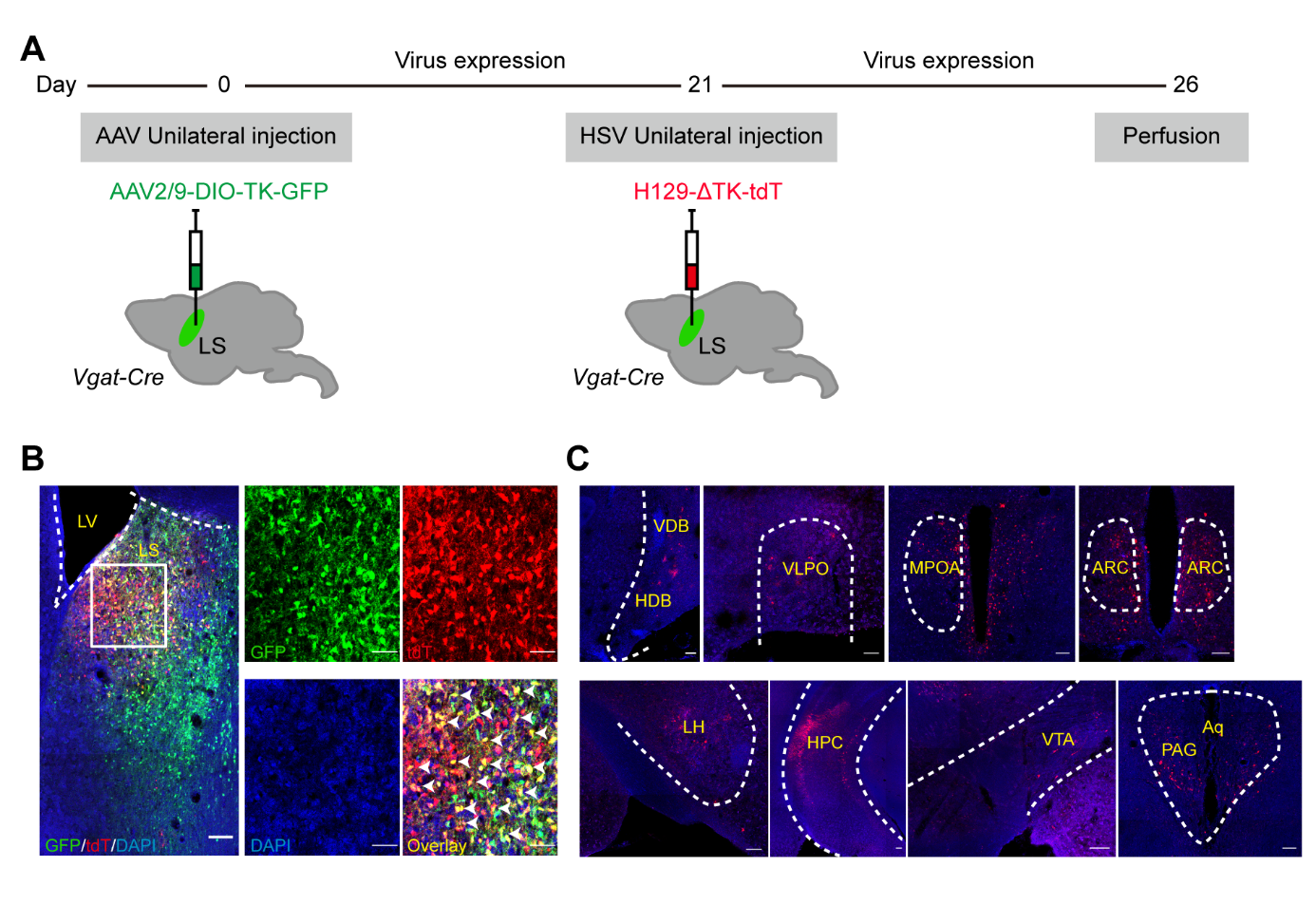
**Fig. S7. Tracing LS GABAergic neurons pathway with H129-ΔTK-tdT.** (**A**) Anterograde tracing of the axons of LS GABAergic neurons with the H129-ΔTK-tdT virus. Experimental timeline for tracing the lateral LS with the helper (AAV-DIO-TK-GFP, day 0) and H129-ΔTK-tdT (day 21). The brains were obtained on day 26. (**B**) Schematics of virus injection and representative images of the labeled starter neurons indicated in LS. The scale bar in LS represents 100 μm and 20 μm, respectively. (**C**) tdTomato-positive cells in VDB, HDB, VLPO, MPOA, LH, ARC, HPC, VTA, and PAG. Scale bar, 100 μm.


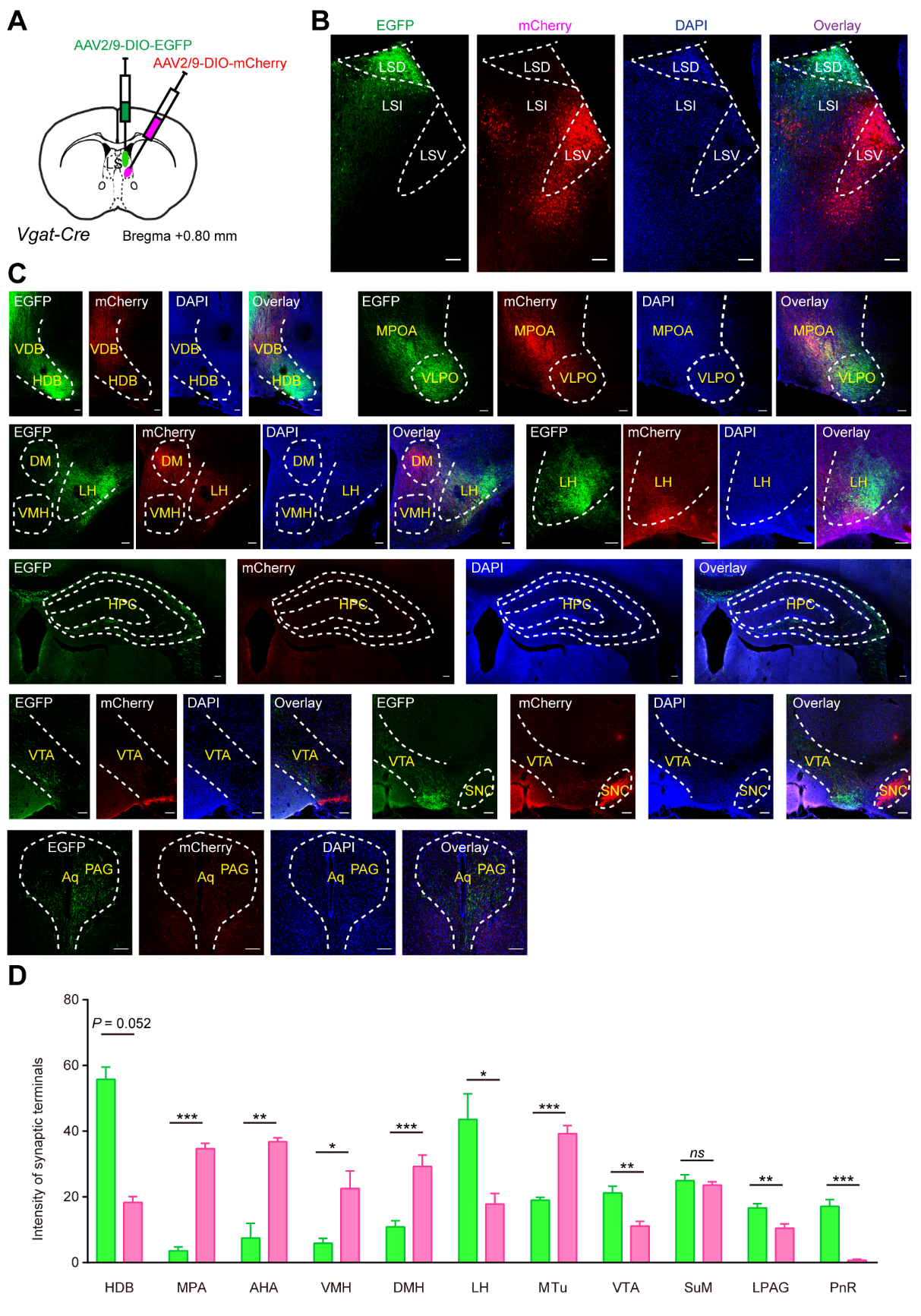
**Fig. S8. Distinct patterns of brain-wide efferent projections from dorsal and ventral LS.** (**A**) Schematics of viral injection: EGFP vector for anterograde tracing was injected into dorsal LS; mCherry was injected into ventral LS. (**B**) A representative image showing that EGFP and mCherry were expressed in dorsal and ventral LS, respectively. Scale bar, 100 μm. (**C**) The distribution pattern of collateral projection fibers anterogradely traced from dorsal and ventral LS GABAergic neurons in VDB, HDB, VLPO, MPOA, LH, DM, VMH, HPC, VTA, SNC, and PAG. Scale bar, 100 μm. (**D**) Comparison of the downstream projections from dorsal versus ventral LS. **P* < 0.05, ***P* < 0.01, ****P* < 0.001, *ns*, no significant difference (*P* > 0.05). Data are presented as the means ± SEM. For further details of statistical data analysis see Table S1.


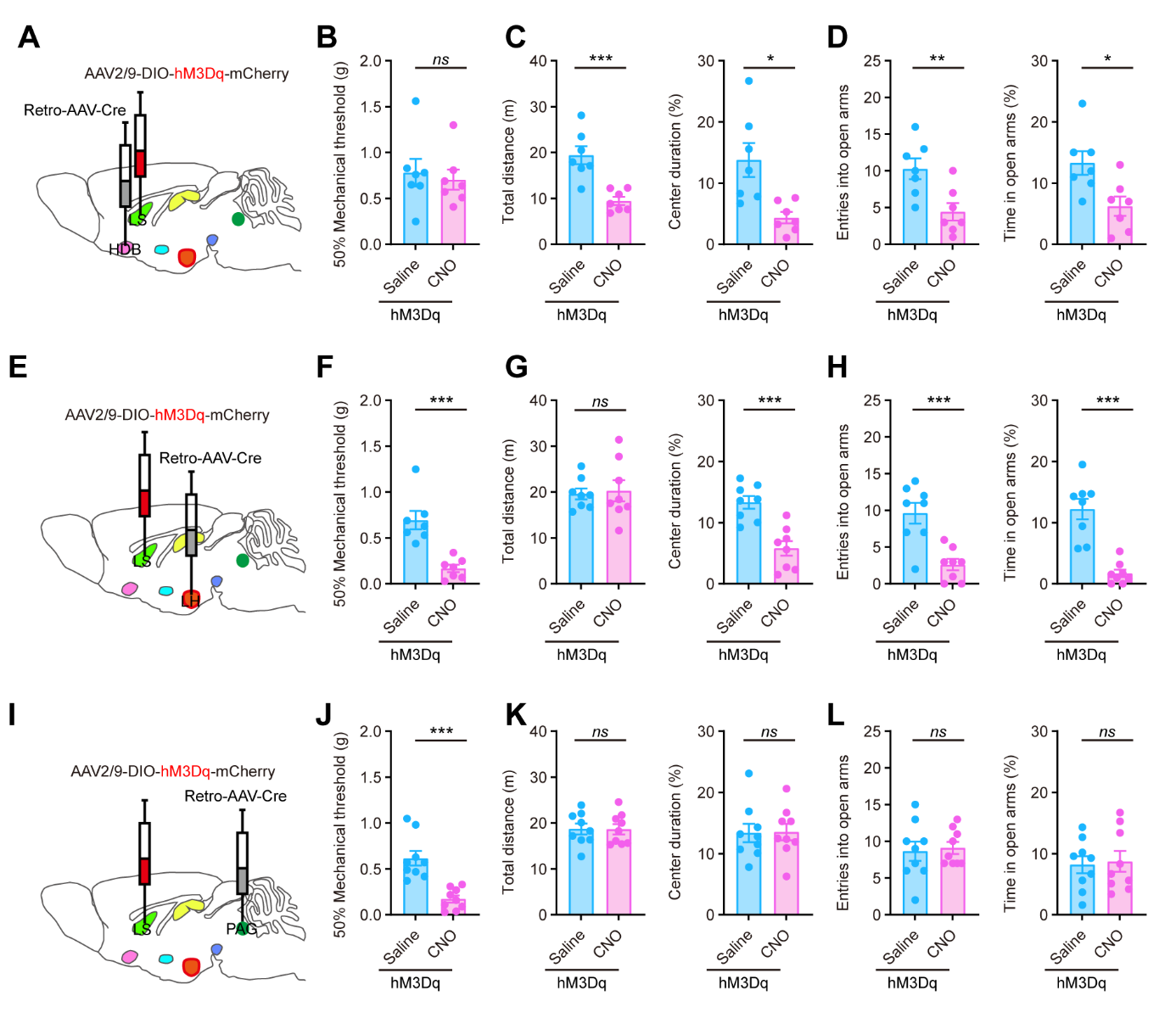
**Fig. S9. Effects of chemogenetic activation of LS-HDB, LS-LH, and LS-PAG circuits on behavioral effects.** (**A, E, I**) The illustration showed injection of Retro-AAV-Cre into the HDB, LH or PAG and AAV2/9-DIO-hM3Dq-mCherry into the LS. (**B-D**) Effects of chemogenetic activation of the HDB-projecting LS neurons on mechanical pain threshold (**B**), (Left) total distance traversed in the OFT, (Right) time spent in central of OFT (**C**), and (Left) total entries in arms, (Right) time spent in open arms (**D**). (**F**-**H**) and (**J-L**) data presented similarly to (**B-D**). **P* < 0.05, ***P* < 0.01, ****P* < 0.001. *ns*, no significant difference (*P* > 0.05). Data are presented as the means ± SEM. For further details of statistical data analysis see Table S1.


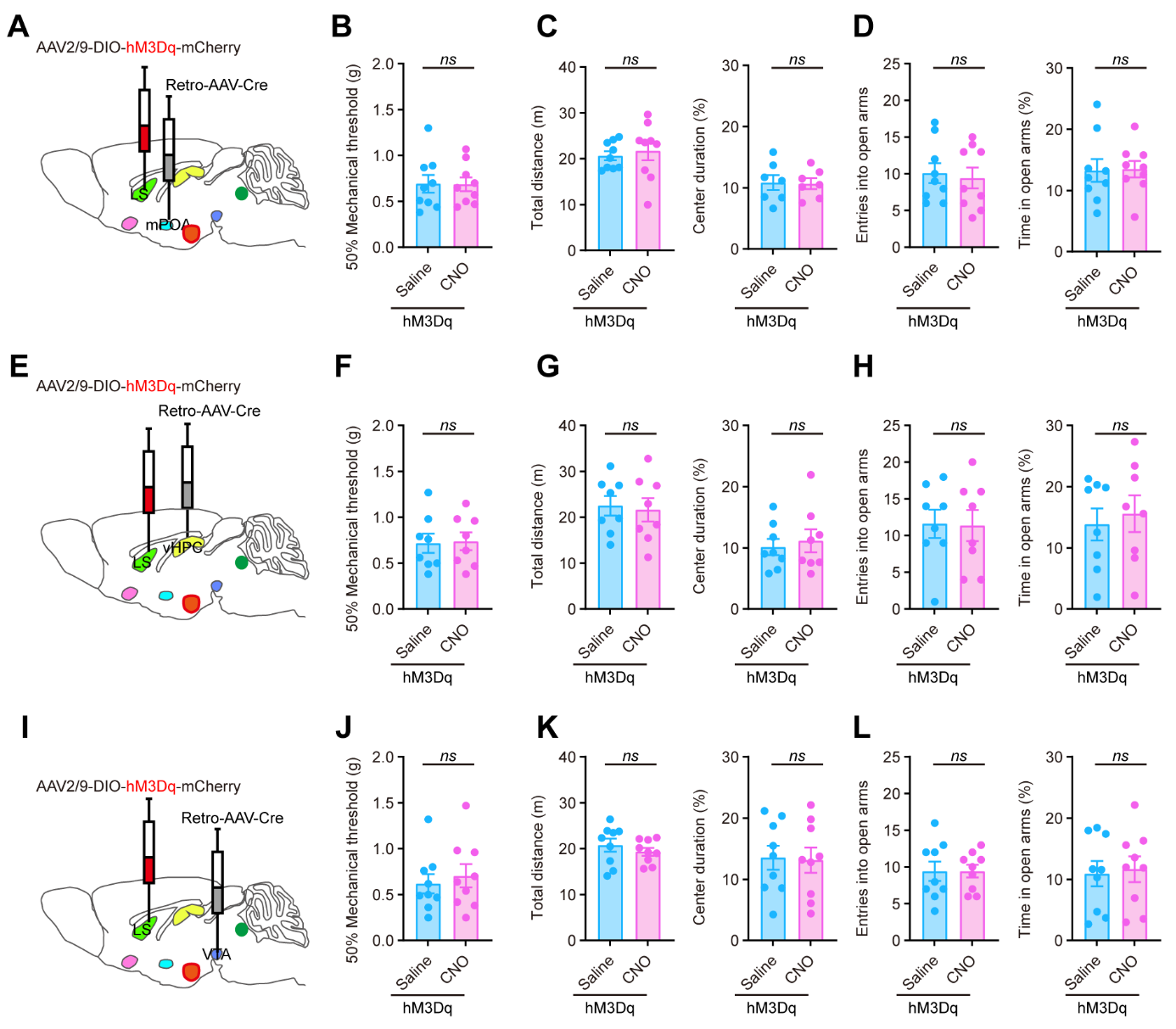
**Fig. S10. Effects of chemogenetic activation of LS-mPOA, LS-vHPC, and LS-VTA circuits on behavioral effects.** (**A, E, I**) The illustration showed injection of Retro-AAV-Cre into the mPOA, vHPC or VTA and AAV2/9-DIO-hM3Dq-mCherry into the LS. (**B-D**) Effects of chemogenetic activation of the mPOA-projecting LS neurons on mechanical pain threshold (**B**), (Left) total distance traversed in the OFT, (Right) time spent in central of OFT (**C**), and (Left) total entries in arms, (Right) time spent in open arms (**D**). (**F**-**H**) and (**J-L**) data presented similarly to (**B-D**). *ns*, no significant difference (*P* > 0.05). Data are presented as the means ± SEM. For further details of statistical data analysis see Table S1.


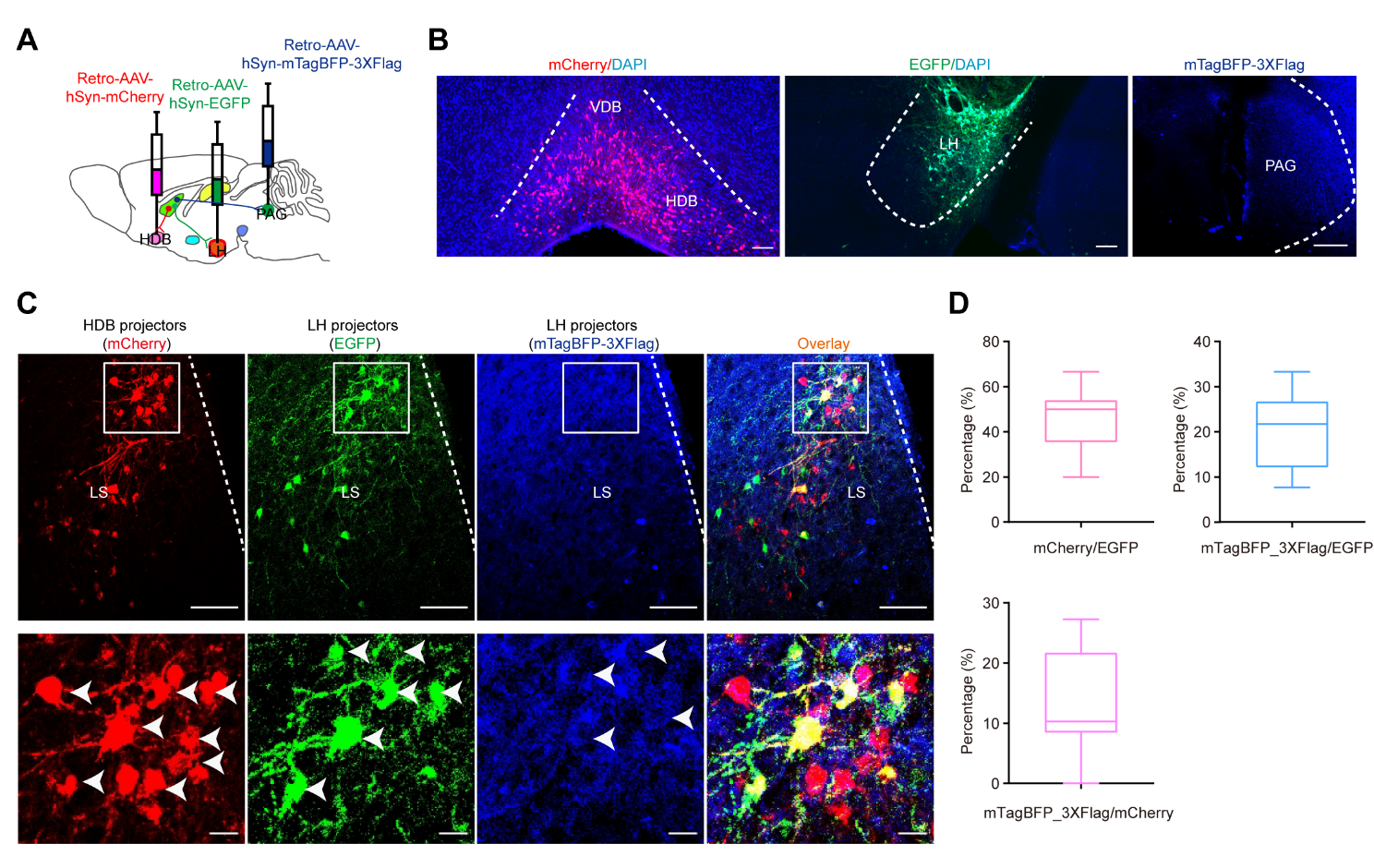
**Fig. S11. LS neurons that project to HDB, LH, and PAG are distinct subpopulations.** (**A**) Schematics of viral injection for simultaneous retrograde tracing from HDB, LH, and PAG. (**B**) Injection sites in HDB, LH, and PAG. Scale bar, 100 μm. (**C**) Fluorescence images of LS neurons expressing EGFP, mCherry, and BFP. The scale bar in LS represents 100 μm and 20 μm, respectively. (**D**) Percentage of neurons co-expressing two tags.


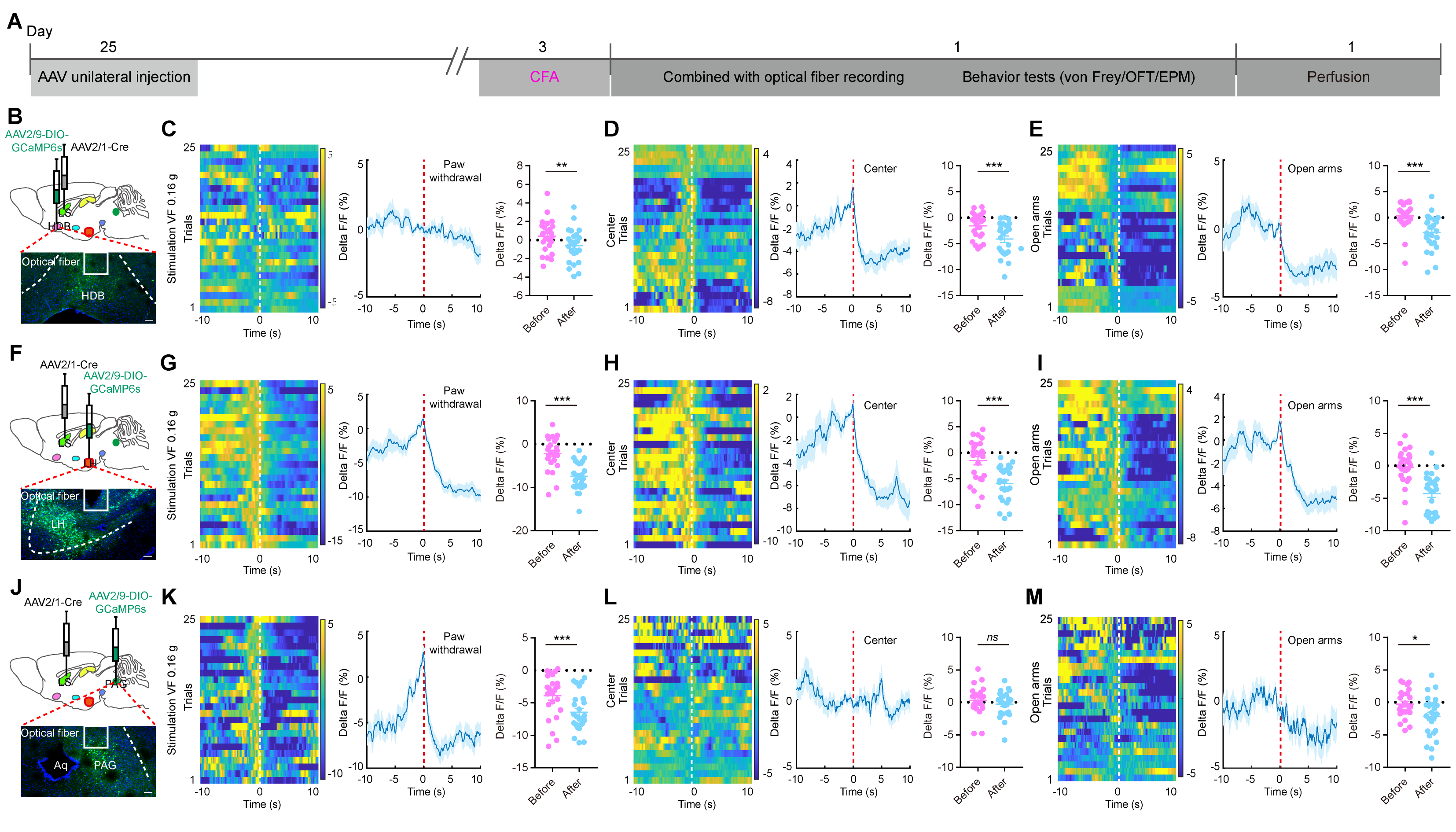


**Fig. S12. Nocifensive and anxiety-like behaviors are associated with the dynamics of LS-HDB, LS-LH and LS-PAG neurons.** (**A**) Experimental timeline. (**B**) Schematic of the brain surgery targeting LS to HDB projections; fiber photometry setup; and GCaMP6s expression in HDB neurons. (**C**) (Left) Dynamics of GCamp6s signal 10 s before and 10 s after the mice receiving 0.16 g von Frey stimulation, (Middle) related averaged GCamp6s deltaF/F each 10 s before and after receiving stimulation, (Right) statistics of the average Ca^2+^ fluorescence signaling changes within a 10-s bin ( -5 to +5 s) of 0.16 g von Frey stimulation (**D**) (Left) Dynamics of GCamp6s signal 10 s before and 10 s after the mice entering the center in OFT, (Middle) and related averaged GCamp6s deltaF/F each 10 s before and after entering the center, and (right) statistics of the average Ca2+ fluorescence signaling changes within a 10-s bin ( -5 to +5 s) before and during entering open arms. (**E**) (Left) Fiber photometry measurement of dynamics calcium signals within a 20 s bin (−10 to +10 s) on the moment the animal crossed from an EPM closed arms to open arms, (Middle) average deltaF/F, before and after the procedures, and (Right) Statistical analysis results. (**F-I**) Same as (**B**-**E**) but targeting the LS to LH projections. Scale bar, 100 μm. (**J-M**) Same as (**B**-**E**) but targeting the LS to PAG projections. **P* < 0.05, ***P* < 0.01, ****P* < 0.001. *ns*, no significant difference (*P* > 0.05). Data are presented as the means ± SEM. For further details of statistical data analysis see Table S1.


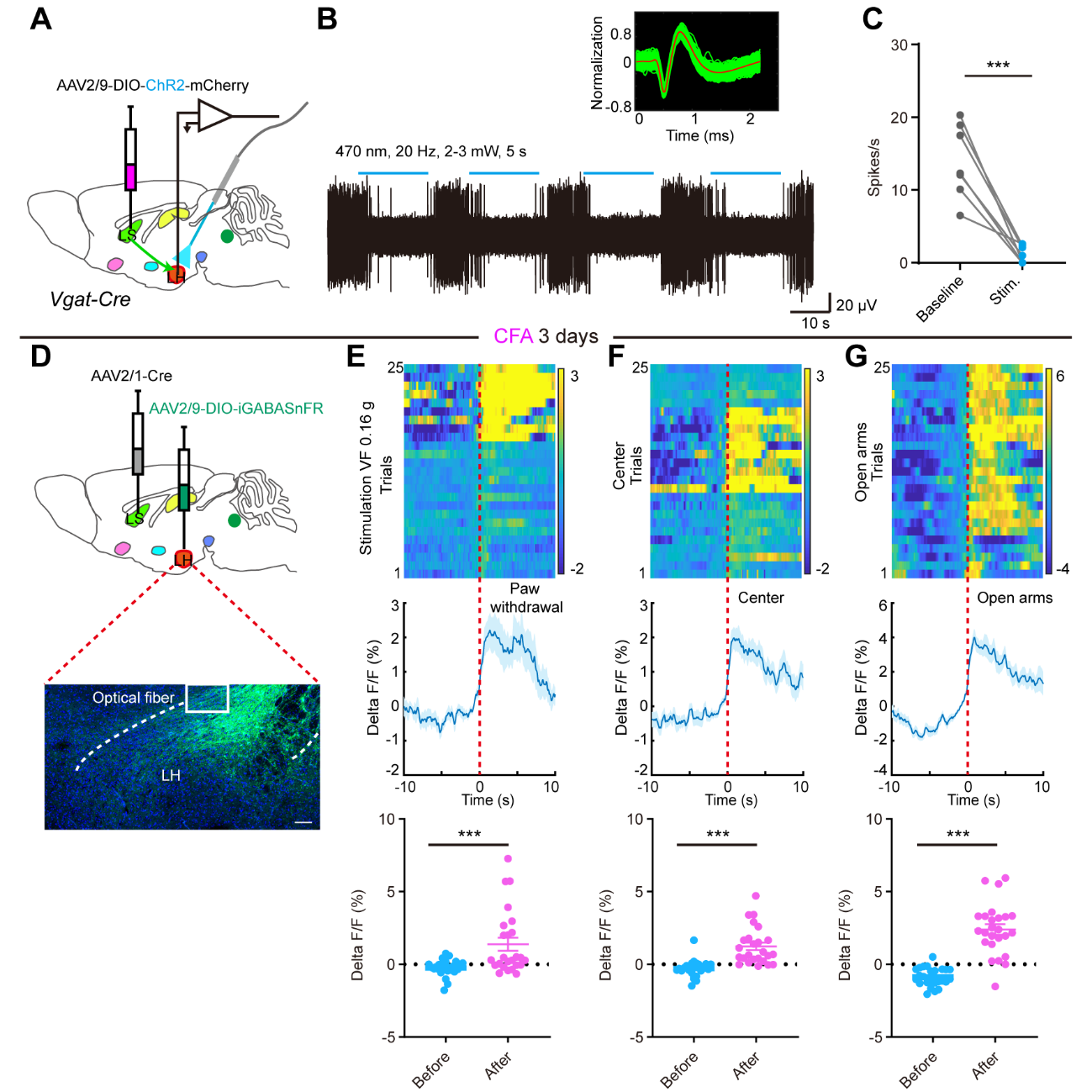
**Fig. S13. Dissection of the LS→LH circuit.** (**A**) Schematics of AAV2/9-DIO–ChR2–mCherry injection and optical fiber placement into LH. Both optogenetic stimulation in axonal terminal of LS GABAergic neurons and electrophysiological recordings in LH were simultaneously performed in vivo. (**B**) (Top) Standardized extracellular action potential (spike) waveform recorded in vivo for an isolated single unit in LH. (Bottom) In vivo electrophysiological recordings of LH single unit firing shows that optogenetic activation of LS GABAergic neurons reliably inhibits neuronal activity in LH. (**C**) Averaged spike rate responses of LH neurons upon ChR2-optogenetic manipulation in LH. (**D**) The illustration showed injection of AAV2/1-Cre into the LS and a genetically encoded GABA sensor (AAV2/9-DIO-iGABASnFR) into LH, and the detection of GABA release in LH from behaviorally manipulated LS neurons. Scale bar, 100 μm. Pain and anxiety promote GABA release from LS-LH circuit: (**E-G**) Heatmap, average and statistical analysis GABA sensor deltaF/F ratios before and during of LS GABAergic neurons in mice receiving von Frey punctate (E), entering the central zone (F), and entering the EPM open arms from closed arms. ****P* < 0.001. Data are presented as the means ± SEM. For further details of statistical data analysis see Table S1.


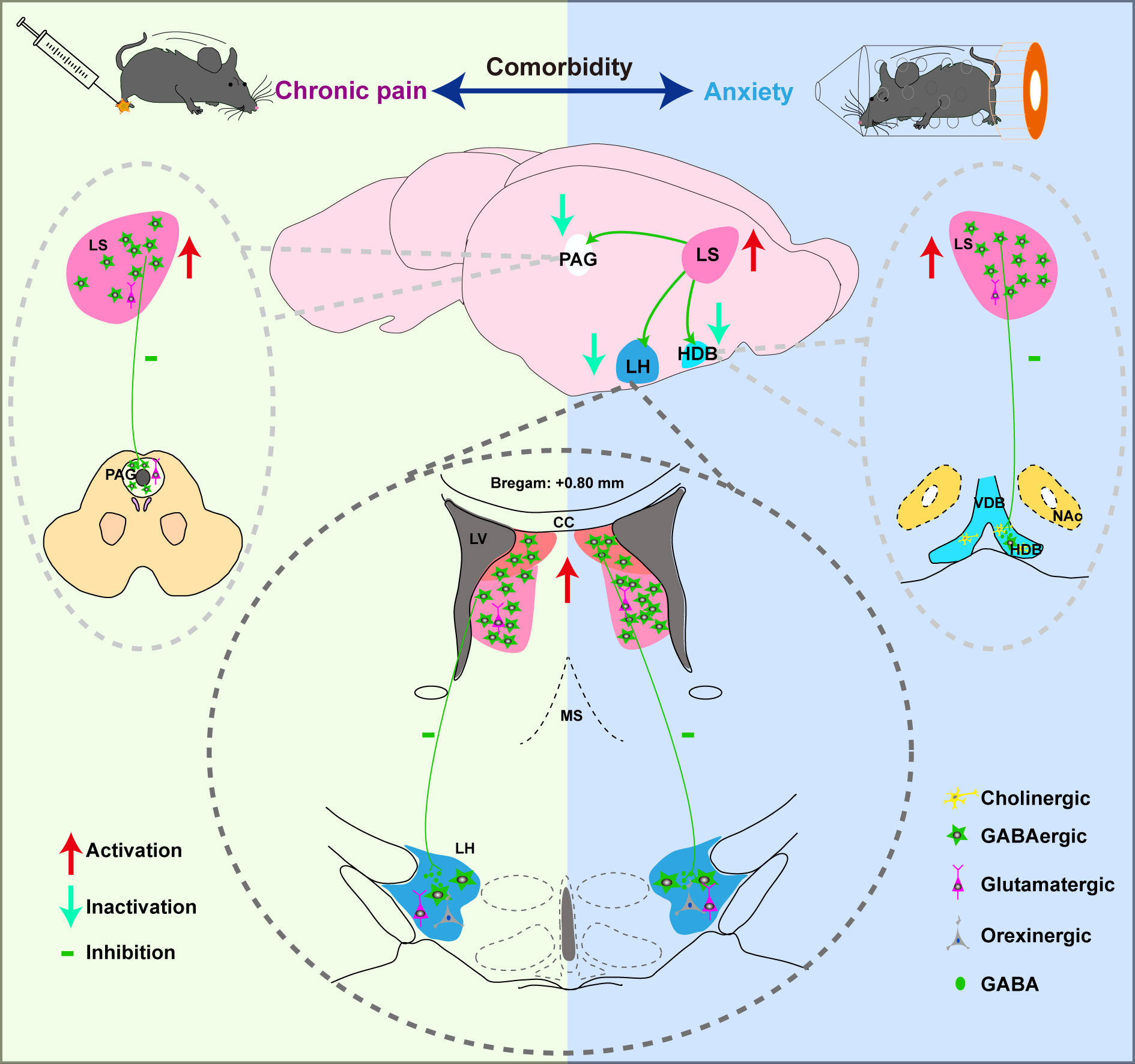


**Fig. S14. The role of LS and its downstream projection pathway regulating hyperalgesia and anxiety behaviors.** Both pain and anxiety activated GABAergic neurons in the LS (red arrow). In turn, the inhibition of LS projection targets (PAG, LH, HDB) was enhanced. The PAG-projecting GABAergic neurons of LS are involved in the regulation of nociception (left ellipse). The GABAergic LS to HDB projection mainly regulates anxiety behaviors (right ellipse). The LS to LH projection regulates pain-anxiety comorbidities (middle ellipse).

**4. References**

37. K. Koga *et al.*, Coexistence of two forms of LTP in ACC provides a synaptic mechanism for the interactions between anxiety and chronic pain. *Neuron* **85**, 377-389 (2015).

38. G. Q. Wang *et al.*, Deactivation of excitatory neurons in the prelimbic cortex via Cdk5 promotes pain sensation and anxiety. *Nat Commun* **6**, 7660 (2015).

39. S. Chiba *et al.*, Chronic restraint stress causes anxiety- and depression-like behaviors, downregulates glucocorticoid receptor expression, and attenuates glutamate release induced by brain-derived neurotrophic factor in the prefrontal cortex. *Prog Neuropsychopharmacol Biol Psychiatry* **39**, 112-119 (2012).

40. S. R. Chaplan, F. W. Bach, J. W. Pogrel, J. M. Chung, T. L. Yaksh, Quantitative assessment of tactile allodynia in the rat paw. *J Neurosci Methods* **53**, 55-63 (1994).

41. H. Wang *et al.*, Incerta-thalamic Circuit Controls Nocifensive Behavior via Cannabinoid Type 1 Receptors. *Neuron* **107**, 538-551 e537 (2020).

42. D. Wang *et al.*, GABAergic Neurons in the Dorsal-Intermediate Lateral Septum Regulate Sleep-Wakefulness and Anesthesia in Mice. *Anesthesiology* **135**, 463-481 (2021).

43 Z. Zhang *et al.*, Neuronal ensembles sufficient for recovery sleep and the sedative actions of alpha2 adrenergic agonists. *Nat Neurosci* **18**, 553-561 (2015).

44. S. Ren *et al.*, The paraventricular thalamus is a critical thalamic area for wakefulness. *Science* **362**, 429-434 (2018).
