# Supplementary material for: Lateral septum-lateral hypothalamus circuit dysfunction in comorbid pain and anxiety
Source: Mol Psychiatry. 2023 Jan 16;28(3):1090–100. doi: 10.1038/s41380-022-01922-y (PMC10005966; doi:10.1038/s41380-022-01922-y)
Supplement: Supplementary file 2 — Table S1 [file 41380_2022_1922_MOESM2_ESM.docx]

**Table S1 Extended statistical information for Figure 1 to 5 and Figure S1 to S13**

| **Figures** | **n** | **Normality** | **Equality of Variances** | **Analysis methods** | **Statistics** | ***P* Value** | **Post hoc multiple comparison tests** |
| --- | --- | --- | --- | --- | --- | --- | --- |
| Figure 1A | n = 13 | No | No | Mann-Whitney rank sum test (two-tailed) | U = 10 | <0.001 |  |
| Figure 1B-left | n = 13 | Yes | Yes | Unpaired t test (two-tailed) | *t* = 0.103 | 0.919 |  |
| Figure 1B-middle | n = 13 | Yes | Yes | Unpaired t test (two-tailed) | *t* = 6.356 | <0.001 |  |
| Figure 1B-right | n = 13 | Yes | Yes | Pearson correlation | *r^2^* = 0.506 | 0.006 |  |
| Figure 1C-left | n = 13 | Yes | Yes | Unpaired t test (two-tailed) | *t* = 2.600 | 0.016 |  |
| Figure 1C-middle | n = 13 | Yes | Yes | Unpaired t test (two-tailed) | *t* = 3.512 | 0.002 |  |
| Figure 1C-right | n = 13 | Yes | Yes | Pearson correlation | *r^2^* = 0.561 | 0.003 |  |
| Figure 1D | n = 21 (from 7 mice) | Yes | No | Unpaired t test with Welch’s correction (two-tailed) | *t* = 14.574 | < 0.001 |  |
| Figure 1E | n = 15 (Control); n = 16 (CRS) | Yes | Yes | Unpaired t test (two-tailed) | *t* = 2.627 | 0.014 |  |
| Figure 1F-left | n = 15 (Control); n = 16 (CRS) | Yes | Yes | Unpaired t test (two-tailed) | *t* = 3.720 | <0.001 |  |
| Figure 1F-middle | n = 15 (Control); n = 16 (CRS) | Yes | No | Unpaired t test with Welch’s correction (two-tailed) | *t* = 5.134 | <0.001 |  |
| Figure 1F-right | n = 16 | Yes | Yes | Pearson correlation | *r^2^* = 0.517 | 0.001 |  |
| Figure 1G left | n = 15 (Control); n = 16 (CRS) | Yes | Yes | Unpaired t test (two-tailed) | *t* = 3.710 | <0.001 |  |
| Figure 1G- middle | n = 15 (Control); n = 16 (CRS) | Yes | Yes | Unpaired t test (two-tailed) | *t* = 5.224 | <0.001 |  |
| Figure 1G-right | n = 16 | Yes | Yes | Pearson correlation | *r^2^* = 0.377 | 0.011 |  |
| Figure 1H | n = 15 (from 5 mice) | Yes | Yes | Unpaired t test (two-tailed) | *t* = 7.346 | <0.001 |  |
| Figure 2C | n = 25 (from 5 mice) | No | No | Kruskal Wallis One Way ANOVA on ranks with Uncorrected Dunn's test | H_2_ = 27.279 | <0.001 | Saline+Gs v.s. CFA+Gs: *P* = 0.001 CFA+GFP v.s. CFA+Gs: *P* < 0.001 |
| Figure 2E | n = 25 (from 5 mice) | No | No | Kruskal Wallis One Way ANOVA on ranks with Uncorrected Dunn's test | H_2_ = 48.976 | <0.001 | Saline+GS v.s. CFA+Gs: *P* < 0.001 CFA+GFP v.s. CFA+Gs: *P* < 0.001 |
| Figure 2G | n = 25 (from 5 mice) | No | No | Kruskal Wallis One Way ANOVA on ranks with Uncorrected Dunn's test | H_2_ = 31.774 | <0.001 | Control+GS v.s. CFA+Gs: *P* = 0.003 CFA+GFP v.s. CFA+Gs: *P* < 0.001 |
| Figure 2I | n = 25 (from 5 mice) | No | No | Kruskal Wallis One Way ANOVA on ranks with Uncorrected Dunn's test | H_2_ = 29.890 | <0.001 | Control+GS v.s. CFA+Gs: *P* < 0.001 CFA+GFP v.s. CFA+Gs: *P* < 0.001 |
| Figure 3C | n = 12 | No | No | Wilcoxon matched-pairs signed rank test | W = 78.000 | <0.001 |  |
| Figure 3D | n = 7 (mCherry); n =8 (ChR2) | Yes | Yes | Two-way RM ANOVA with LSD post hoc comparison | F(1, 52) = 6.827 | 0.012 | left: on (mCherry v.s. ChR2): *P* = 0.029 right: on (mCherry v.s. ChR2): *P* = 0.008 |
| Figure 3F-left | n = 6 (mCherry); n = 7 (ChR2) | Yes | Yes | Two-way RM ANOVA with LSD post hoc comparison | F(1, 22) = 0.002 | 0.963 | Off (mCherry) v.s. on (mCherry): *P* = 0.592  off (mCherry) v.s. off (ChR2): *P* = 0.945 off (mCherry) v.s. on (ChR2): *P* = 0.673 off (ChR2) v.s. on (ChR2): *P* = 0.609 |
| Figure 3F-right | n = 6 (mCherry); n = 7 (ChR2) | Yes | Yes | Two-way RM ANOVA with LSD post hoc comparison | F(1, 22) = 5.429 | 0.029 | Off (mCherry) v.s. on (mCherry): *P* = 0.905  off (mCherry) v.s. off (ChR2): *P* = 0.589 off (mCherry) v.s. on (ChR2): *P* = 0.001 off (ChR2) v.s. on (ChR2): *P* = 0.003 |
| Figure 3H-left | n = 6 (mCherry); n = 7 (ChR2) | Yes | Yes | Two-way RM ANOVA with LSD post hoc comparison | F(1, 22) = 5.076 | 0.003 | Off (mCherry) v.s. on (mCherry): *P* = 0.855  off (mCherry) v.s. off (ChR2): *P* = 0.626 off (mCherry) v.s. on (ChR2): *P* < 0.001  off (ChR2) v.s. on (ChR2): *P* = 0.002 |
| Figure 3H-right | n = 6 (mCherry); n = 7 (ChR2) | Yes | Yes | Two-way RM ANOVA with LSD post hoc comparison | F(1, 22) = 4.725 | 0.041 | Off (mCherry) v.s. on (mCherry): *P* = 0.865  off (mCherry) v.s. off (ChR2): *P* = 0.685 off (mCherry) v.s. on (ChR2): *P* = 0.009 off (ChR2) v.s. on (ChR2): *P* = 0.003 |
| Figure 3K | n = 6 | Yes | Yes | paired t test (two-tailed) | *t* = 7.001 | <0.001 |  |
| Figure3L | n = 9 | Yes | Yes | Two-way RM ANOVA with LSD post hoc comparison | F(3, 160) = 5.043 | 0.002 | **Baseline:**  mCherry+Saline v.s. hM3Dq+CNO: *P* = 0.869 mCherry+CNO v.s. hM3Dq+CNO: *P* = 0.712  hM3Dq+Saline v.s. hM3Dq+CNO: *P* = 0.844 **0.5 h:**  mCherry+Saline v.s. hM3Dq+CNO: *P* < 0.001  mCherry+CNO v.s. hM3Dq+CNO: *P* < 0.001 hM3Dq+Saline v.s. hM3Dq+CNO: *P* = 0.006 **1.5 h:**  mCherry+Saline v.s. hM3Dq+CNO: *P* = 0.025  mCherry+CNO v.s. hM3Dq+CNO: *P* = 0.009  hM3Dq+Saline v.s. hM3Dq+CNO: *P* = 0.079 **2.5 h:**  mCherry+Saline v.s. hM3Dq+CNO: *P* = 0.258  mCherry+CNO v.s. hM3Dq+CNO: *P* = 0.235 hM3Dq+Saline v.s. hM3Dq+CNO: *P* = 0.426 **4 h:**  mCherry+Saline v.s. hM3Dq+CNO: *P* = 0.736 mCherry+CNO v.s. hM3Dq+CNO: *P* = 0.294 hM3Dq+Saline v.s. hM3Dq+CNO: *P* = 0.793 |
| Figure 3N-left | n = 9 | Yes | Yes | Two-way RM ANOVA with LSD post hoc comparison | F(1, 32) = 0.072 | 0.790 | Saline (mCherry) v.s. CNO: (mCherry): *P* = 0.404  Saline (mCherry) v.s. Saline (hM3Dq): *P* = 0.945  Saline (mCherry) v.s. CNO (hM3Dq): *P* = 0.256 Saline (hM3Dq) v.s. CNO (hM3Dq): *P* = 0.229 |
| Figure 3N-right | n = 9 | Yes | Yes | Two-way RM ANOVA with LSD post hoc comparison | F(1, 32) = 10.070 | 0.003 | Saline (mCherry) v.s. CNO (mCherry): *P* = 0.987 Saline (mCherry) v.s. Saline (hM3Dq): *P* = 0.987 Saline (mCherry) v.s. CNO (hM3Dq): *P* < 0.001  Saline (hM3Dq) v.s. CNO (hM3Dq): *P* < 0.001 |
| Figure 3P-left | n = 9 | Yes | Yes | Two-way RM ANOVA with LSD post hoc comparison | F(1, 32) = 6.919 | 0.013 | Saline (mCherry) v.s. CNO (mCherry): *P* = 0.889 Saline (mCherry) v.s. Saline (hM3Dq): *P* = 0.523 Saline (mCherry) v.s. CNO (hM3Dq): *P* < 0.001  Saline (hM3Dq) v.s. CNO (hM3Dq): *P* = 0.001 |
| Figure 3P-right | n = 9 | Yes | Yes | Two-way RM ANOVA with LSD post hoc comparison | F(1, 32) = 6.557 | 0.015 | Saline (mCherry) v.s. CNO (mCherry): *P* = 0.909 Saline (mCherry) v.s. Saline (hM3Dq): *P* = 0.857 Saline (mCherry) v.s. CNO (hM3Dq): *P* = 0.002  Saline (hM3Dq) v.s. CNO (hM3Dq): *P* = 0.001 |
| Figure 4C | n =10 | Yes | Yes | paired t test (two-tailed) | *t* = 4.555 | 0.001 |  |
| Figure 4D | n = 8 | Yes | Yes | Two-way RM ANOVA with LSD post hoc comparison | F(1, 56) = 39.030 | < 0.001 | left, on (mCherry v.s. NpHR): *P* < 0.001 right, on (mCherry v.s. NpHR): *P* < 0.001 |
| Figure 4F-left | n = 8 | Yes | Yes | Two-way RM ANOVA with LSD post hoc comparison | F(1, 28) = 0.328 | 0.572 | off (mCherry) v.s. on (mCherry): *P* = 0.705 off (mCherry) v.s. off (NpHR): *P* = 0.589  off (mCherry) v.s. on (NpHR): *P* = 0.906 off (NpHR) v.s. on (NpHR): *P* = 0.673 |
| Figure 4F-right | n = 8 | Yes | Yes | Two-way RM ANOVA with LSD post hoc comparison | F(1, 28) = 7.006 | 0.013 | off (mCherry) v.s. on (mCherry): *P* = 0.836 off (mCherry) v.s. off (NpHR): *P* = 0.553  off (mCherry) v.s. on (NpHR): *P* < 0.001 off (NpHR) v.s. on (NpHR): *P* = 0.002 |
| Figure 4H-left | n = 8 | Yes | Yes | Two-way RM ANOVA with LSD post hoc comparison | F(1, 28) = 16.170 | < 0.001 | off (mCherry) v.s. on (mCherry): *P* > 0.999 off (mCherry) v.s. off (NpHR): *P* = 0.523  off (mCherry) v.s. on (NpHR): *P* < 0.001 off (NpHR) v.s. on (NpHR): *P* < 0.001 |
| Figure 4H-right | n = 8 | Yes | Yes | Two-way RM ANOVA with LSD post hoc comparison | F(1, 28) = 6.004 | 0.021 | off (mCherry) v.s. on (mCherry): *P* = 0.754 off (mCherry) v.s. off (NpHR): *P* = 0.886  off (mCherry) v.s. on (NpHR): *P* = 0.001 off (NpHR) v.s. on (NpHR): *P* < 0.001 |
| Figure 4I | n = 8 | Yes | Yes | Two-way RM ANOVA with LSD post hoc comparison | F(1, 56) = 18.270 | < 0.001 | left, on (mCherry v.s. NpHR): *P* < 0.001  right, on (mCherry v.s. NpHR): *P* < 0.001 |
| Figure 4K-left | n = 8 | Yes | Yes | Two-way RM ANOVA with LSD post hoc comparison | F (1, 28) = 3.490 | 0.072 | off (mCherry) v.s. on (mCherry): *P* = 0.935 off (mCherry) v.s. off (NpHR): *P* = 0.765  off (mCherry) v.s. on (NpHR): *P* = 0.005 off (NpHR) v.s. on (NpHR): *P* = 0.011 |
| Figure 4K-right | n = 8 | Yes | Yes | Two-way RM ANOVA with LSD post hoc comparison | F(1, 28) = 19.430 | < 0.001 | off (mCherry) v.s. on (mCherry): *P* = 0.646 off (mCherry) v.s. off (NpHR): *P* = 0.747  off (mCherry) v.s. on (NpHR): *P* < 0.001 off (NpHR) v.s. on (NpHR): *P* < 0.001 |
| Figure 4M-left | n = 8 | Yes | Yes | Two-way RM ANOVA with LSD post hoc comparison | F(1, 28) = 8.130 | 0.008 | off (mCherry) v.s. on (mCherry): *P* = 0.795 off (mCherry) v.s. off (NpHR): *P* = 0.729  off (mCherry) v.s. on (NpHR): *P* < 0.001 off (NpHR) vs. on (NpHR): *P* < 0.001 |
| Figure 4M-right | n = 8 | Yes | Yes | Two-way RM ANOVA with LSD post hoc comparison | F(1, 28) = 14.700 | < 0.001 | off (mCherry) v.s. on (mCherry): *P* = 0.835 off (mCherry) v.s. off (NpHR): *P* = 0.723  off (mCherry) v.s. on (NpHR): *P* < 0.001 off (NpHR) v.s. on (NpHR): *P* < 0.001 |
| Figure 5E | n = 7 (Saline); n =8 (CNO) | Yes | Yes | Unpaired t test (two-tailed) | *t* = 0.222 | 0.828 |  |
| Figure 5F-left | n = 7 (Saline); n =8 (CNO) | Yes | Yes | Unpaired t test (two-tailed) | *t* = 3.536 | 0.004 |  |
| Figure 5F-right | n = 7 (Saline); n =8 (CNO) | Yes | Yes | Unpaired t test (two-tailed) | *t* = 3.155 | 0.008 |  |
| Figure 5G-left | n = 7 (Saline); n =8 (CNO) | Yes | Yes | Unpaired t test (two-tailed) | *t* = 2.037 | 0.062 |  |
| Figure 5G-right | n = 7 (Saline); n =8 (CNO) | Yes | Yes | Unpaired t test (two-tailed) | *t* = 2.127 | 0.053 |  |
| Figure 5I | n = 7 (Saline); n = 9 (CNO) | Yes | No | Unpaired t test with Welch’s correction (two-tailed) | *t* = 3.390 | 0.011 |  |
| Figure 5J-left | n = 7 (Saline); n = 9 (CNO) | Yes | Yes | Unpaired t test (two-tailed) | *t* = 4.083 | 0.001 |  |
| Figure 5J-right | n = 7 (Saline); n = 9 (CNO) | Yes | Yes | Unpaired t test (two-tailed) | *t* = 3.532 | 0.003 |  |
| Figure 5K-left | n = 7 (Saline); n = 9 (CNO) | Yes | Yes | Unpaired t test (two-tailed) | *t* = 2.825 | 0.014 |  |
| Figure 5K-right | n = 7 (Saline); n = 9 (CNO) | Yes | Yes | Unpaired t test (two-tailed) | *t* = 3.527 | 0.003 |  |
| Figure 5M | n = 8 (Saline); n = 10 (CNO) | Yes | No | Unpaired t test with Welch’s correction (two-tailed) | *t* = 3.338 | 0.009 |  |
| Figure 5N-left | n = 8 (Saline); n = 10 (CNO) | Yes | Yes | Unpaired t test (two-tailed) | *t* = 0.398 | 0.696 |  |
| Figure 5N-right | n = 8 (Saline); n = 10 (CNO) | Yes | Yes | Unpaired t test (two-tailed) | *t* = 0.511 | 0.616 |  |
| Figure 5O-left | n = 8 (Saline); n = 10 (CNO) | Yes | Yes | Unpaired t test (two-tailed) | *t* = 0.178 | 0.861 |  |
| Figure 5O-right | n = 8 (Saline); n = 10 (CNO) | Yes | Yes | Unpaired t test (two-tailed) | *t* = 0.286 | 0.779 |  |
| Figure S1A-left | n = 13 | Yes | Yes | Unpaired t test (two-tailed) | *t* = 0.044 | 0.965 |  |
| Figure S1A-right | n = 13 | Yes | Yes | Unpaired t test (two-tailed) | *t* = 0.235 | 0.816 |  |
| Figure S1B-left | n = 15 (Control); n = 16 (CRS) | Yes | Yes | Unpaired t test (two-tailed) | *t* = 0.369 | 0.715 |  |
| Figure S1B-right | n = 15 (Control); n = 16 (CRS) | Yes | Yes | Unpaired t test (two-tailed) | *t* = 0.936 | 0.357 |  |
| Figure S2A | n = 15 | No | Yes | Mann-Whitney rank sum test (two-tailed) | U = 64.500 | 0.047 |  |
| Figure S2B | n = 15 (Control); n = 16 (CRS) | Yes | Yes | Unpaired t test (two-tailed) | *t* = 4.869 | <0.001 |  |
| Figure S3E | n = 25 (from 5 mice) | No | No | Kruskal Wallis One Way ANOVA on ranks with Uncorrected Dunn's test | H_2_ = 37.150 | <0.001 | Saline+GS v.s. CFA+Gs: *P* = 0.023 CFA+GFP v.s. CFA+Gs: *P* < 0.001 |
| Figure S3G | n = 25 | No | No | Kruskal Wallis One Way ANOVA on ranks with Uncorrected Dunn's test | H_2_ = 41.060 | <0.001 | Saline+GS v.s. CFA+Gs: *P* < 0.001 CFA+GFP v.s. CFA+Gs: *P* < 0.001 |
| Figure S4C-left | n = 10 | Yes | Yes | Two-way RM ANOVA with LSD post hoc comparison | F (1, 36) = 15.310 | < 0.001 | Pre- (Saline) v.s. Pre- (CNO): *P* = 0.770 Pre-(Saline) v.s. Post- (Saline): *P* = 0.898  Pre-(Saline) v.s. Post- (CNO): *P* < 0.001 Post- (Saline) v.s. Post- (CNO): *P* < 0.001 |
| Figure S4C-right | n = 10 | Yes | Yes | Two-way RM ANOVA with LSD post hoc comparison | F (1, 36) = 4.556 | 0.039 | Pre- (Saline) v.s. Pre- (CNO): *P* = 0.635  Pre-(Saline) v.s. Post- (Saline): *P* = 0.519  Pre-(Saline) v.s. Post- (CNO): *P* = 0.007  Post- (Saline) v.s. Post- (CNO): *P* = 0.001 |
| Figure S4D-left | n = 10 | Yes | Yes | Two-way RM ANOVA with LSD post hoc comparison | F (1, 36) = 0.475 | 0.495 | Pre- (Saline) v.s. Pre- (CNO): *P* = 0.927  Pre-(Saline) v.s. Post- (Saline): *P* = 0.491  Pre-(Saline) v.s. Post- (CNO): *P* = 0.713  Post- (Saline) v.s. Post- (CNO): *P* = 0.293 |
| Figure S4D-right | n = 10 | Yes | Yes | Two-way RM ANOVA with LSD post hoc comparison | F (1, 36) = 4.368 | 0.044 | Pre- (Saline) v.s. Pre- (CNO): *P* = 0.532  Pre-(Saline) v.s. Post- (Saline): *P* = 0.589  Pre-(Saline) v.s. Post- (CNO): *P* = 0.004  Post- (Saline) v.s. Post- (CNO): *P*< 0.001 |
| Figure S4E-Baseline | n = 10 | No | Yes | Mann-Whitney rank sum test (two-tailed) | U = 44 | 0.671 |  |
| Figure S4E-Pre-injection | n = 9 (Saline); n =10 (CNO) | Yes | Yes | Unpaired t test (two-tailed) | *t* = 0.011 | 0.992 |  |
| Figure S4E-0.5 h | n = 9 (Saline); n =10 (CNO) | Yes | Yes | Unpaired t test (two-tailed) | *t* = 3.043 | 0.007 |  |
| Figure S4E-1.5 h | n = 9 (Saline); n =10 (CNO) | Yes | Yes | Unpaired t test (two-tailed) | *t* = 1.742 | 0.099 |  |
| Figure S4E-2.5 h | n = 9 (Saline); n =10 (CNO) | Yes | Yes | Unpaired t test (two-tailed) | *t* = 1.090 | 0.291 |  |
| Figure S4E-4 h | n = 9 (Saline); n =10 (CNO) | Yes | Yes | Unpaired t test (two-tailed) | *t* = 0.161 | 0.874 |  |
| Figure S5C | n = 6 | Yes | Yes | Unpaired t test (two-tailed) | *t* = 3.110 | 0.011 |  |
| Figure S5D | n = 8 (Control mCherry); n = 7 (taCasp3) | Yes | Yes | Two-way RM ANOVA with LSD post hoc comparison | F(4, 65) = 7.638 | <0.001 | day 7: *P* < 0.001  day 14: *P* < 0.001 day 21: *P* < 0.001 |
| Figure S5E- left | n = 8 | Yes | Yes | Two-way RM ANOVA with LSD post hoc comparison | F(4, 70) = 0.732 | 0.573 | day 7: *P* = 0.506 day 14: *P* = 0.201 day 21: *P* = 0.408 |
| Figure S5E-right | n = 8 | Yes | Yes | Two-way RM ANOVA with LSD post hoc comparison | F(4, 70) = 1.342 | 0.263 | day7: *P* = 0.015 day14: *P* = 0.019 day21: *P* = 0.123 |
| Figure S5F-left | n = 8 | Yes | Yes | Two-way RM ANOVA with LSD post hoc comparison | F (4, 70) = 3.078 | 0.022 | day7: *P* = 0.002 day14: *P* < 0.001 day21: *P* < 0.001 |
| Figure S5F-right | n = 8 | Yes | Yes | Two-way RM ANOVA with LSD post hoc comparison | F (4, 70) = 2.917 | 0.027 | day7: *P* < 0.001 day14: *P* < 0.001 day21: *P* < 0.001 |
| Figure S5G | n = 27 (from 9 mice) | Yes | Yes | Pearson correlation | *r^2^* = 0.685 | < 0.001 |  |
| Figure S5H | n = 23 (from 5 mice) | Yes | Yes | Pearson correlation | *r^2^* = 0.594 | < 0.001 |  |
| Figure S5J-left | n = 8 | Yes | Yes | Unpaired t test (two-tailed) | *t* = 2.830 | 0.013 |  |
| Figure S5J-right | n = 8 | Yes | No | Unpaired t test with Welch’s correction (two-tailed) | *t* = 4.916 | 0.001 |  |
| Figure S5K-left | n = 8 | Yes | Yes | Unpaired t test (two-tailed) | *t* = 6.142 | < 0.001 |  |
| Figure S5K-right | n = 8 | Yes | Yes | Unpaired t test (two-tailed) | *t* = 4.222 | < 0.001 |  |
| Figure S6B | n = 6 | Yes | Yes | paired t test (two-tailed) | *t* = 7.507 | <0.001 |  |
| Figure S6C | n = 11 | Yes | Yes | Two-way RM ANOVA with LSD post hoc comparison | F(12, 200) = 6.348 | < 0.001 | **Baseline:**  mCherry+Saline v.s. hM4Di+CNO: *P* = 0.206  mCherry+CNO v.s. hM4Di+CNO: *P* = 0.537 hM4Di+Saline v.s. hM4Di+CNO: *P* = 0.612 **0.5 h:**  mCherry+Saline v.s. hM4Di+CNO: *P* < 0.001 mCherry+CNO v.s. hM4Di+CNO: *P* < 0.001 hM4Di+Saline v.s. hM4Di+CNO: *P* < 0.001 **1.5 h:**  mCherry+Saline v.s. hM4Di+CNO: *P* < 0.001 mCherry+CNO v.s. hM4Di+CNO: *P* < 0.001 hM4Di+Saline v.s. hM4Di+CNO: *P* < 0.001 **2.5 h:**  mCherry+Saline v.s. hM4Di+CNO: *P* = 0.006  mCherry+CNO v.s. hM4Di+CNO: *P* = 0.041 hM4Di+Saline v.s. hM4Di+CNO: *P* = 0.006 **4 h:**  mCherry+Saline v.s. hM4Di+CNO: *P* = 0.949 mCherry+CNO v.s. hM4Di+CNO: *P* = 0.419 hM4Di+Saline v.s. hM4Di+CNO: *P* = 0.912 |
| Figure S6E-left | n = 11 | Yes | Yes | Two-way RM ANOVA with LSD post hoc comparison | F(1, 40) = 6.739 | 0.013 | Saline (mCherry) v.s. CNO (mCherry): *P* = 0.806 Saline (mCherry) v.s. Saline (hM4Di): *P* = 0.424 Saline (mCherry) v.s. CNO (hM4Di): *P* < 0.001 Saline (hM4Di) v.s. CNO (hM4Di): *P* < 0.001 |
| Figure S6E-right | n = 11 (mCherry); n = 8 (hM3Dq) | Yes | Yes | Two-way RM ANOVA with LSD post hoc comparison | F(1, 34) = 2.852 | 0.100 | Saline (mCherry) v.s. CNO (mCherry): *P* = 0.324 Saline (mCherry) v.s. Saline (hM4Di): *P* = 0.256 Saline (mCherry) v.s. CNO (hM4Di): *P* < 0.001 Saline (hM4Di) v.s. CNO (hM4Di): *P* = 0.004 |
| Figure S6G-left | n = 11 | Yes | Yes | Two-way RM ANOVA with LSD post hoc comparison | F(1, 40) = 4.437 | 0.042 | Saline (mCherry) v.s. CNO (mCherry): *P* = 0.685 Saline (mCherry) v.s. Saline (hM4Di): *P* = 0.919 Saline (mCherry) v.s. CNO (hM4Di): *P* = 0.014 Saline (hM4Di) v.s. CNO (hM4Di): *P* = 0.014 |
| Figure S6G- right | n = 11 | Yes | Yes | Two-way RM ANOVA with LSD post hoc comparison | F(1, 40) = 9.086 | 0.005 | Saline (mCherry) v.s. CNO (mCherry): *P* = 0.995 Saline (mCherry) v.s. Saline (hM4Di): *P* = 0.923 Saline (mCherry) v.s. CNO (hM4Di): *P* < 0.001 Saline (hM4Di) v.s. CNO (hM4Di): *P* < 0.001 |
| Figure S6H | n = 11 (mCherry); n = 13 (hM4Di) | Yes | Yes | Two-way RM ANOVA with LSD post hoc comparison | F(12, 220) = 2.491 | 0.004 | **Baseline:**  mCherry+Saline v.s. hM4Di+CNO: *P* = 0.948  mCherry+CNO v.s. hM4Di+CNO: *P* = 0.872 hM4Di+Saline v.s. hM4Di+CNO: *P* = 0.549 **0.5 h:**  mCherry+Saline v.s. hM4Di+CNO: *P* < 0.001 mCherry+CNO v.s. hM4Di+CNO: *P* < 0.001 hM4Di+Saline v.s. hM4Di+CNO: *P* < 0.001 **1.5 h:**  mCherry+Saline v.s. hM4Di+CNO: *P* = 0.017 mCherry+CNO v.s. hM4Di+CNO: *P* = 0.008 hM4Di+Saline v.s. hM4Di+CNO: *P* = 0.037 **2.5 h:**  mCherry+Saline v.s. hM4Di+CNO: *P* = 0.635 mCherry+CNO v.s. hM4Di+CNO: *P* = 0.387 hM4Di+Saline v.s. hM4Di+CNO: *P* = 0.535 **4 h:**  mCherry+Saline v.s. hM4Di+CNO: *P* = 0.642 mCherry+CNO v.s. hM4Di+CNO: *P* = 0.829 hM4Di+Saline v.s. hM4Di+CNO: *P* = 0.983 |
| Figure S6J-left | n = 11 (mCherry) n = 13 (hM4Di) | Yes | Yes | Two-way RM ANOVA with LSD post hoc comparison | F(1, 44) = 15.460 | < 0.001 | Saline (mCherry) v.s. CNO (mCherry): *P* = 0.721 Saline (mCherry) v.s. Saline (hM4Di): *P* = 0.402 Saline (mCherry) v.s. CNO (hM4Di): *P* < 0.001 Saline (hM4Di) v.s. CNO (hM4Di): *P* < 0.001 |
| Figure S6J-right | n = 11 (mCherry) n = 13 (hM4Di) | Yes | Yes | Two-way RM ANOVA with LSD post hoc comparison | F(1, 44) = 6.823 | 0.012 | Saline (mCherry) v.s. CNO (mCherry): *P* = 0.862 Saline (mCherry): v.s. Saline (hM4Di): *P* = 0.899 Saline (mCherry) v.s. CNO (hM4Di): *P* = 0.002 Saline (hM4Di) v.s. CNO (hM4Di): *P* < 0.001 |
| Figure S6L-left | n = 11 (mCherry) n = 13 (hM4Di) | Yes | Yes | Two-way RM ANOVA with LSD post hoc comparison | F(1, 44) = 7.009 | 0.011 | Saline (mCherry) v.s. CNO (mCherry): *P* = 0.755 Saline (mCherry) v.s. Saline (hM4Di): *P* = 0.947 Saline (mCherry) v.s. CNO (hM4Di): *P* = 0.002 Saline (hM4Di) v.s. CNO (hM4Di): *P* < 0.001 |
| Figure S6L-right | n = 11 (Cherry)  n = 14 (hM4Di) | Yes | Yes | Two-way RM ANOVA with LSD post hoc comparison | F(1, 46) = 9.494 | 0.004 | Saline (mCherry) v.s. CNO (mCherry): *P* = 0.927 Saline (mCherry) v.s. Saline (hM4Di): *P* = 0.614 Saline (mCherry) v.s. CNO (hM4Di): *P* < 0.001 Saline (hM4Di) v.s. CNO (hM4Di): *P* < 0.001 |
| Figure S8D-HBD | n = 5 (EGFP)  n = 6 (mCherry) | No | Yes | Mann Whitney Rank Sum Test (two-tailed) | U = 4 | 0.052 |  |
| Figure S8D-MPA | n = 5 | Yes | Yes | Unpaired t test (two-tailed) | *t* = 15.130 | < 0.001 |  |
| Figure S8D-AHA | n = 5 | No | No | Mann Whitney Rank Sum Test (two-tailed) | U = 0 | 0.008 |  |
| Figure S8D-VMH | n = 6 | Yes | No | Unpaired t test with Welch’s correction (two-tailed) | *t* = 3.036 | 0.024 |  |
| Figure S8D-DMH | n = 8 | Yes | Yes | Unpaired t test (two-tailed) | *t* = 4.647 | < 0.001 |  |
| Figure S8D-LH | n = 7 | Yes | No | Unpaired t test with Welch’s correction (two-tailed) | *t* = 3.043 | 0.016 |  |
| Figure S8D-Mtu | n = 5 | Yes | Yes | Unpaired t test (two-tailed) | *t* = 7.738 | < 0.001 |  |
| Figure S8-D VTA | n = 5 | Yes | Yes | Unpaired t test (two-tailed) | *t* = 4.126 | 0.003 |  |
| Figure S8D-SuM | n = 5 | Yes | Yes | Unpaired t test (two-tailed) | *t* = 0.622 | 0.551 |  |
| Figure S8D-PAG | n = 7 | Yes | Yes | Unpaired t test (two-tailed) | *t* = 3.376 | 0.006 |  |
| Figure S8D-PnR | n = 4 | Yes | Yes | Unpaired t test with Welch’s correction (two-tailed) | *t* = 12.818 | < 0.001 |  |
| Figure S9B | n = 7 | No | Yes | Mann-Whitney rank sum test (two-tailed) | U = 18.500 | 0.476 |  |
| Figure S9C-left | n = 7 | Yes | Yes | Unpaired t test (two-tailed) | *t* = 4.595 | <0.001 |  |
| Figure S9C-right | n = 7 | Yes | No | Unpaired t test with Welch’s correction (two-tailed) | *t* = 3.239 | 0.013 |  |
| Figure S9D-left | n = 7 | Yes | Yes | Unpaired t test (two-tailed) | *t* = 3.173 | 0.008 |  |
| Figure S9D-right | n = 7 | Yes | Yes | Unpaired t test (two-tailed) | *t* = 2.827 | 0.015 |  |
| Figure S9F | n = 7 | No | No | Mann-Whitney rank sum test (two-tailed) | U = 0 | <0.001 |  |
| Figure S9G-left | n = 8 | Yes | Yes | Unpaired t test (two-tailed) | *t* = 0.264 | 0.796 |  |
| Figure S9G-right | n = 8 | Yes | Yes | Unpaired t test (two-tailed) | *t* = 4.715 | <0.001 |  |
| Figure S9H-left | n = 8 | Yes | Yes | Unpaired t test (two-tailed) | *t* = 4.339 | <0.001 |  |
| Figure S9H-right | n = 8 | Yes | No | Unpaired t test with Welch’s correction (two-tailed) | *t* = 5.839 | <0.001 |  |
| Figure S9J | n = 9 | Yes | Yes | Unpaired t test (two-tailed) | *t* = 5.002 | <0.001 |  |
| Figure S9K-left | n = 9 | Yes | Yes | Unpaired t test (two-tailed) | *t* = 0.041 | 0.968 |  |
| Figure S9K-right | n = 9 | Yes | Yes | Unpaired t test (two-tailed) | *t* = 0.083 | 0.935 |  |
| Figure S9L-left | n = 9 | Yes | Yes | Unpaired t test (two-tailed) | *t* = 0.289 | 0.777 |  |
| Figure S9L-right | n = 9 | Yes | Yes | Unpaired t test (two-tailed) | *t* = 0.255 | 0.802 |  |
| Figure S10B | n = 9 | Yes | Yes | Unpaired t test (two-tailed) | *t* = 0.054 | 0.957 |  |
| Figure S10C-left | n = 9 | Yes | Yes | Unpaired t test (two-tailed) | *t* = 0.470 | 0.645 |  |
| Figure S10C-right | n = 7 | Yes | Yes | Unpaired t test (two-tailed) | *t* = 0.119 | 0.908 |  |
| Figure S10D-left | n = 9 | Yes | Yes | Unpaired t test (two-tailed) | *t* = 0.337 | 0.740 |  |
| Figure S10D-right | n = 9 | Yes | Yes | Unpaired t test (two-tailed) | *t* = 0.097 | 0.924 |  |
| Figure S10F | n = 8 | Yes | Yes | Unpaired t test (two-tailed) | *t* = 0.157 | 0.878 |  |
| Figure S10G-left | n = 8 | Yes | Yes | Unpaired t test (two-tailed) | *t* = 0.264 | 0.795 |  |
| Figure S10G-right | n = 8 | Yes | Yes | Unpaired t test (two-tailed) | *t* = 0.444 | 0.664 |  |
| Figure S10H-left | n = 8 | Yes | Yes | Unpaired t test (two-tailed) | *t* = 0.087 | 0.932 |  |
| Figure S10H-right | n = 8 | Yes | Yes | Unpaired t test (two-tailed) | *t* = 0.439 | 0.668 |  |
| Figure S10J | n = 9 | Yes | Yes | Unpaired t test (two-tailed) | *t* = 0.520 | 0.610 |  |
| Figure S10K-left | n = 9 | Yes | Yes | Unpaired t test (two-tailed) | *t* = 0.852 | 0.407 |  |
| Figure S10K-right | n = 9 | Yes | Yes | Unpaired t test (two-tailed) | *t* = 0.142 | 0.889 |  |
| Figure S10L-left | n = 9 | Yes | Yes | Unpaired t test (two-tailed) | *t* = 0.000 | >0.999 |  |
| Figure S10L-right | n = 9 | Yes | Yes | Unpaired t test (two-tailed) | *t* = 0.244 | 0.810 |  |
| Figure S12C | n = 25 (from 5 mice) | Yes | Yes | paired t test (two-tailed) | *t* = 3.252 | 0.003 |  |
| Figure S12D | n = 25 (from 5 mice) | No | Yes | Wilcoxon matched-pairs signed rank test | W = -299 | < 0.001 |  |
| Figure S12E | n = 25 (from 5 mice) | No | Yes | Wilcoxon matched-pairs signed rank test | W = -317 | < 0.001 |  |
| Figure S12G | n = 25 (from 5 mice) | Yes | Yes | paired t test (two-tailed) | *t* = 9.410 | < 0.001 |  |
| Figure S12H | n = 25 (from 5 mice) | No | Yes | Wilcoxon matched-pairs signed rank test | W = -315 | < 0.001 |  |
| Figure S12I | n = 25 (from 5 mice) | Yes | Yes | paired t test (two-tailed) | *t* = 5.737 | < 0.001 |  |
| Figure S12K | n = 25 (from 5 mice) | Yes | Yes | paired t test (two-tailed) | *t* = 4.095 | < 0.001 |  |
| Figure S12L | n = 25 (from 5 mice) | No | Yes | Wilcoxon matched-pairs signed rank test | W = -115 | 0.127 |  |
| Figure S12M | n = 25 (from 5 mice) | Yes | Yes | paired t test (two-tailed) | *t* = 2.721 | 0.012 |  |
| Figure S13C | n = 7 | Yes | Yes | paired t test (two-tailed) | *t* = 6.466 | <0.001 |  |
| Figure S13E | n = 25 (from 5 mice) | No | No | Wilcoxon matched-pairs signed rank test | W = 263 | <0.001 |  |
| Figure S13F | n = 25 (from 5 mice) | No | No | Wilcoxon matched-pairs signed rank test | W = 323 | <0.001 |  |
| Figure S13G | n = 25 (from 5 mice) | No | No | Wilcoxon matched-pairs signed rank test | W = 321 | <0.001 |  |
